# Longitudinal associations of diurnal rest-activity rhythms with fatigue, insomnia, and health-related quality of life in survivors of colorectal cancer up to 5 years post-treatment

**DOI:** 10.1186/s12966-024-01601-x

**Published:** 2024-05-02

**Authors:** Marvin Y. Chong, Koen G. Frenken, Simone J. P. M. Eussen, Annemarie Koster, Gerda K. Pot, Stéphanie O. Breukink, Maryska Janssen-Heijnen, Eric T. P. Keulen, Wouter Bijnens, Laurien M. Buffart, Kenneth Meijer, Frank A. J. L. Scheer, Karen Steindorf, Judith de Vos-Geelen, Matty P. Weijenberg, Eline H. van Roekel, Martijn J. L. Bours

**Affiliations:** 1 Present Address: Department of Epidemiology, GROW Research Institute for Oncology and Reproduction, Maastricht University, Maastricht, The Netherlands; 2 Department of Epidemiology, CARIM Cardiovascular Research Institute Maastricht, Maastricht University, Maastricht, The Netherlands; 3 Department of Epidemiology, CAPHRI Care and Public Health Research Institute, Maastricht University, Maastricht, The Netherlands; 4 Department of Social Medicine, CAPHRI Care and Public Health Research Institute, Maastricht University, Maastricht, The Netherlands; 5 Nutrition and Healthcare Alliance, Hospital Gelderse Vallei, Ede, The Netherlands; 6 Department of Surgery, GROW Research Institute for Oncology and Reproduction, NUTRIM Research Institute of Nutrition and Translational Research in Metabolism, Maastricht University Medical Centre+, Maastricht, The Netherlands; 7 Department of Clinical Epidemiology, VieCuri Medical Centre, Venlo, The Netherlands; 8 Department of Internal Medicine and Gastroenterology, Zuyderland Medical Centre Sittard-Geleen, Geleen, The Netherlands; 9 Research Engineering (IDEE), Maastricht University, Maastricht, The Netherlands; 10 Department of Medical BioSciences, Radboud University Medical Center, Nijmegen, The Netherlands; 11 Department of Nutrition and Movement Sciences, NUTRIM Research Institute of Nutrition and Translational Research in Metabolism, Maastricht University Medical Centre+, Maastricht, The Netherlands; 12 Division of Sleep Medicine, Harvard Medical School, Boston, MA USA; 13 Medical Chronobiology Program, Division of Sleep and Circadian Disorders, Departments of Medicine and Neurology, Brigham and Women’s Hospital, Boston, MA USA; 14 Division of Physical Activity, Prevention and Cancer, German Cancer Research Center (DKFZ), Heidelberg, Germany; 15 Department of Internal Medicine, Division of Medical Oncology, GROW Research Institute for Oncology and Reproduction, Maastricht University Medical Centre, Maastricht, The Netherlands

**Keywords:** Colorectal cancer survivorship, Diurnal rest-activity rhythms, Fatigue, Insomnia, Health-related quality of life

## Abstract

**Background:**

There is a growing population of survivors of colorectal cancer (CRC). Fatigue and insomnia are common symptoms after CRC, negatively influencing health-related quality of life (HRQoL). Besides increasing physical activity and decreasing sedentary behavior, the timing and patterns of physical activity and rest over the 24-h day (i.e. diurnal rest-activity rhythms) could also play a role in alleviating these symptoms and improving HRQoL. We investigated longitudinal associations of the diurnal rest-activity rhythm (RAR) with fatigue, insomnia, and HRQoL in survivors of CRC.

**Methods:**

In a prospective cohort study among survivors of stage I-III CRC, 5 repeated measurements were performed from 6 weeks up to 5 years post-treatment. Parameters of RAR, including mesor, amplitude, acrophase, circadian quotient, dichotomy index, and 24-h autocorrelation coefficient, were assessed by a custom MATLAB program using data from tri-axial accelerometers worn on the upper thigh for 7 consecutive days. Fatigue, insomnia, and HRQoL were measured by validated questionnaires. Confounder-adjusted linear mixed models were applied to analyze longitudinal associations of RAR with fatigue, insomnia, and HRQoL from 6 weeks until 5 years post-treatment. Additionally, intra-individual and inter-individual associations over time were separated.

**Results:**

Data were available from 289 survivors of CRC. All RAR parameters except for 24-h autocorrelation increased from 6 weeks to 6 months post-treatment, after which they remained relatively stable. A higher mesor, amplitude, circadian quotient, dichotomy index, and 24-h autocorrelation were statistically significantly associated with less fatigue and better HRQoL over time. A higher amplitude and circadian quotient were associated with lower insomnia. Most of these associations appeared driven by both within-person changes over time and between-person differences in RAR parameters. No significant associations were observed for acrophase.

**Conclusions:**

In the first five years after CRC treatment, adhering to a generally more active (mesor) and consistent (24-h autocorrelation) RAR, with a pronounced peak activity (amplitude) and a marked difference between daytime and nighttime activity (dichotomy index) was found to be associated with lower fatigue, lower insomnia, and a better HRQoL. Future intervention studies are needed to investigate if restoring RAR among survivors of CRC could help to alleviate symptoms of fatigue and insomnia while enhancing their HRQoL.

**Trial registration:**

EnCoRe study NL6904 (https://www.onderzoekmetmensen.nl/).

**Supplementary Information:**

The online version contains supplementary material available at 10.1186/s12966-024-01601-x.

## Background

The incidence of colorectal cancer (CRC) is steadily rising, posing an increasing challenge to public health [1]. At the same time, there has been a notable increase in survival rates, reflecting advancements in early detection due to national screening programs, improved treatments, and comprehensive care strategies, resulting in growing numbers of survivors of CRC [2]. Survivors of CRC often experience a decline in their health-related quality of life (HRQoL), due to symptoms caused by the cancer and/or its treatment [3]. Fatigue and insomnia (sleep problems) are among the most common and debilitating symptoms reported after CRC [4]. Up to two-thirds of survivors of CRC are still affected by one of these distressing symptoms up to three years after diagnosis, further exacerbating the physical and emotional burden on former patients with CRC [4, 5]. Therefore, it is important to alleviate symptoms of fatigue and insomnia during the CRC survivorship period to promote their HRQoL.

Partially shared underlying causes of fatigue and insomnia may include chrono-biological factors influencing daily rhythms, such as the timing, amplitude, and regularity of daily rhythms in physical activity [6, 7]. Multiple physiological processes such as sleep, immune function, and metabolism exhibit cyclic patterns approximating 24 h, referred to as circadian rhythms [8, 9]. These circadian rhythms are primarily entrained by light, through the central circadian clock located in the suprachiasmatic nucleus (SCN), but can also be affected by physical activity and dietary intake through peripheral clocks found in almost all organ systems [9, 10]. Previous research in survivors of CRC showed that less sedentary behavior and more physical activity were associated with lower fatigue and higher HRQoL in the first two years after the end of cancer treatment [11–13]. Besides the intensity and type of physical activity, the timing of physical activity and rest may also be important for perceived fatigue and HRQoL of this population. Appropriately timed diurnal rest-activity rhythms (RAR) may have the potential to restore synchronicity and improve alignment between central and peripheral clocks [10], thereby potentially also decreasing the risk of negative health outcomes such as fatigue and insomnia after cancer.

In previous research, wrist-worn actigraphy has been employed for measuring several parameters encompassing different aspects of RAR in patients with breast cancer and metastatic CRC [14–17]. These RAR parameters were related to mean activity levels (mesor), the contrast between peak- and mean activity levels (amplitude), the timing of the peak activity (acrophase), the difference between daytime and nighttime activity (dichotomy index), and consistency of RAR from one day to the next (24-h autocorrelation coefficient). In these patients, lower values for most of these parameters were observed compared to non-cancer adults, indicating a more disrupted RAR [15–17]. In turn, a more disrupted RAR has been associated with more fatigue, a lower physical and emotional well-being, and a lower quality of life in patients with breast cancer [15, 18, 19] and metastatic CRC [14, 20, 21]. However, longitudinal research on RAR in relation to patient-reported health outcomes among survivors of CRC is scarce. Therefore, we aimed to study longitudinal associations of RAR parameters with fatigue, insomnia, and HRQoL among survivors of stage I-III CRC, using five repeated measurements between 6 weeks and 5 years after CRC treatment. We hypothesized that less disrupted RAR, indicated by higher values for mesor, amplitude, dichotomy index, and 24-h autocorrelation coefficient, are associated with lower fatigue and insomnia, and a higher HRQoL after CRC treatment.

## Methods

### Study design and population

**Fig. 1:**
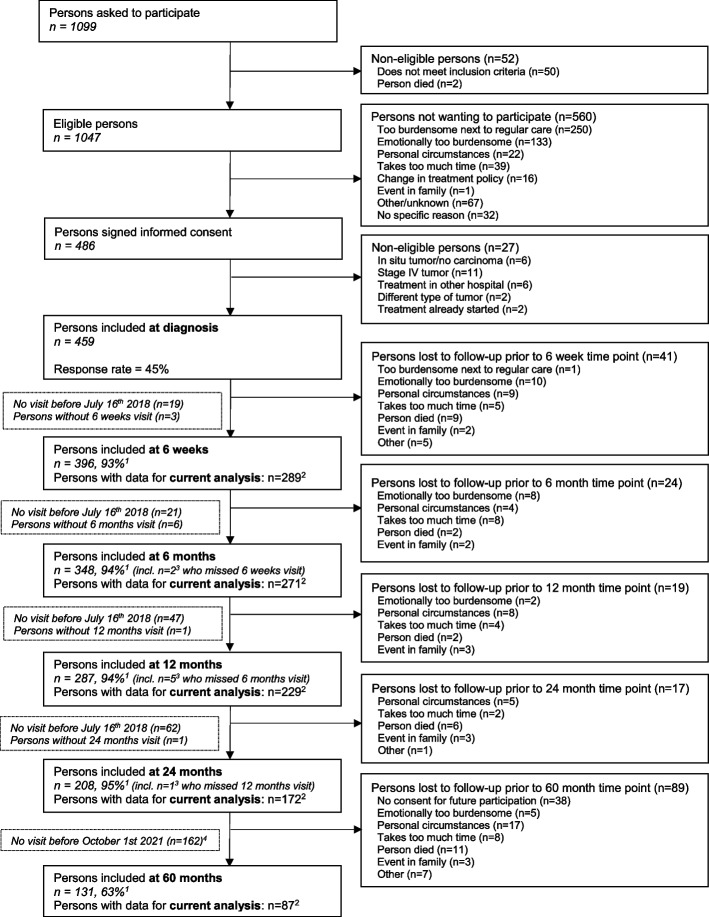
Flow diagram of the inclusion of participants within the Energy for Life after ColoRectal cancer (EnCoRe) study from 2012 onwards and the number of post-treatment measurements available up to October 2021 included in the analyses of the current paper. ^1^Response rate = (persons with measurements)/(persons with measurements + persons lost to follow-up – persons died). The declining number of participants at subsequent time points is because not all participants included at diagnosis from April 2012 onwards had already reached all follow-up time points by July 2018 (6 weeks until 24 months post-treatment) or October 2021 (60 months post-treatment). ^2^Since the analyses in this paper were focused on diurnal rest-activity rhythms and fatigue, insomnia, and health-related quality of life (HRQoL) after colorectal cancer treatment, only post-treatment measurements with available data on diurnal rest-activity rhythms, fatigue, insomnia, or HRQoL, and covariates were included. ^3^Of the three persons without 6 weeks follow-up visits, one person did not have a 6 months follow-up visit before July 16th 2018. Of the six persons without 6 months follow-up visits, one person did not have a 12 months follow-up visit before July 16th 2018. ^4^All data collected from participants between April 2012 and July 2018 were used for the measurements from 6 weeks until 24 months post-treatment. In addition, these participants were followed over time and data was collected in October 2021 for the 60 months-treatment follow-up measurement

We used data of the *En*ergy for Life after *Co*lo*Re*ctal cancer (EnCoRe) study, an ongoing prospective cohort study of survivors of stage I-III CRC in the Netherlands (Netherlands Trial Register number: NL6904) [22]. From April 2012 onwards, all patients diagnosed with stage I-III CRC in three hospitals in the south of the Netherlands (Maastricht University Medical Center + , VieCuri Medical Center, and Zuyderland Medical Center) were eligible for inclusion. Exclusion criteria were stage IV CRC, age below 18 years, not able to understand and speak the Dutch language, a residential address outside of the Netherlands, or one or more comorbidities that could hinder successful participation (e.g., a cognitive disorder, or problems with visibility or hearing). Data were collected by trained dietitians visiting participants during 6 repeated home visits: one at diagnosis before the start of cancer treatment, and five after the end of cancer treatment (surgery, radiotherapy and/or chemotherapy) at 6 weeks, 6 months, 12 months, 24 months, and 60 months post-treatment. From October 2020 onwards, due to the COVID-19 pandemic, data were collected remotely via postal methods. All data collected between April 2012 and July 2018 were used for the present analyses. In addition, data collected until October 2021 were included for the 60-month post-treatment follow-up measurement. The Medical Ethics Committee of the University Hospital Maastricht and Maastricht University approved the study (METC 11–3-075). All patients provided written informed consent prior to participation, and the study was performed in accordance with the Declaration of Helsinki. A flow diagram describing recruitment and follow-up procedures of participants within the EnCoRe study can be found in Fig. 1.

### Diurnal rest-activity rhythm

Parameters of RAR were measured using the validated tri-axial MOX activity monitor (Maastricht Instruments B.V., Maastricht, The Netherlands) [23]. Participants wore the MOX on the anterior upper thigh ten cm above the knee for seven consecutive days (24 h/day) at every post-treatment time point, but not at diagnosis. The MOX measures raw acceleration data in three orthogonal sensor axes at a sampling rate of 25 Hz. These acceleration data were converted into activity counts in 1-min epochs (intervals), using the signal magnitude area [24]. The epochs were used for determining RAR parameters as described below. Monitor wear days with 24 h of wear time were considered valid (i.e. days with non-wear were excluded); only participants with ≥ 4 valid days including at least one weekend day were included in the analyses. Based on these criteria, 4.0% of accelerometer measurements were excluded.

**Fig. 2:**
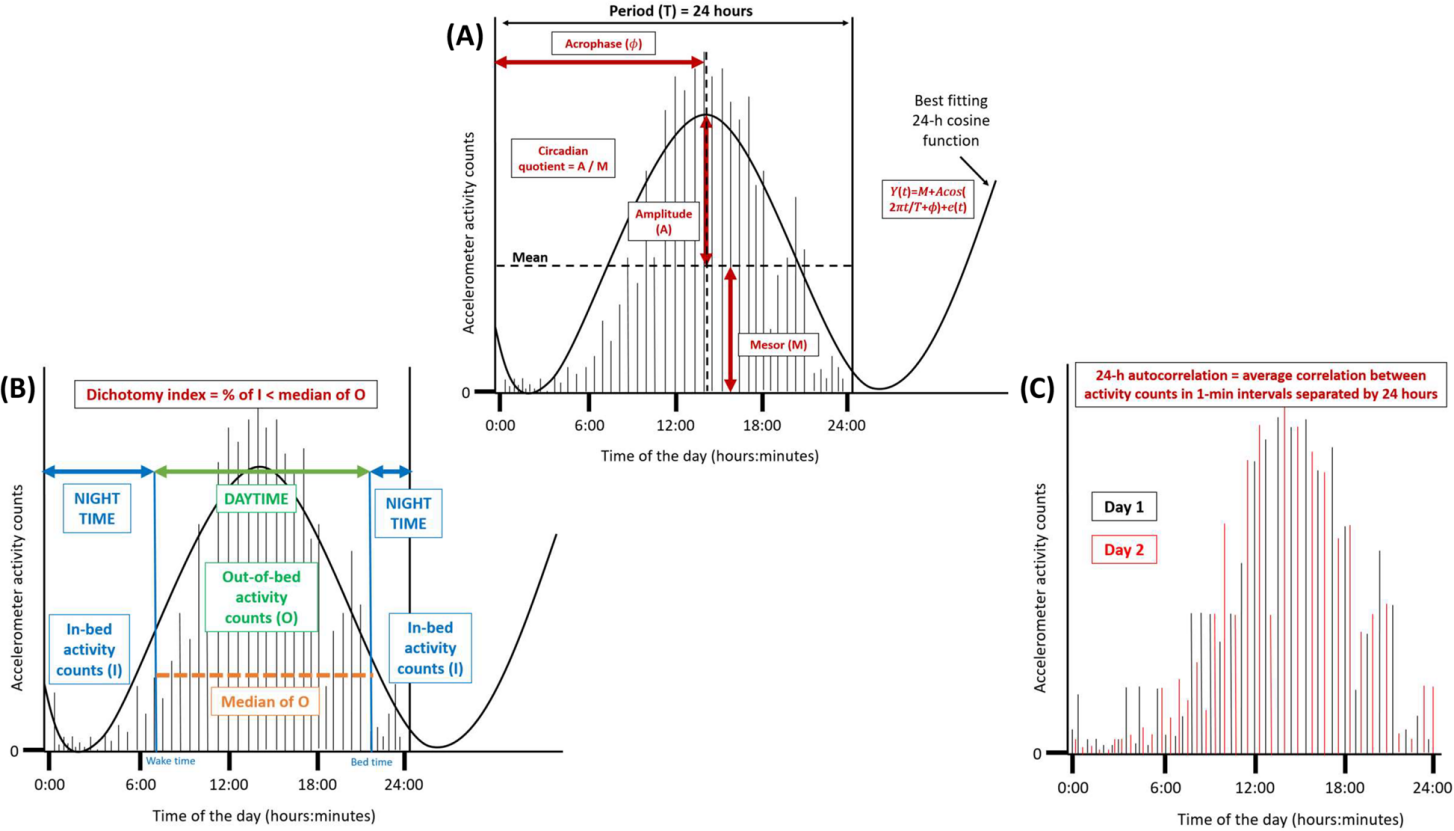
Visualization of the operationalized diurnal rest-activity rhythm parameters: mesor, amplitude, acrophase, circadian quotient, dichotomy index, and 24-h autocorrelation coefficient, based on fictitious accelerometer data. **A** Example of a day the accelerometer was worn by a participant. Activity counts of the participant in each 1-min interval of a 24-h day were available. Based on how these activity counts were distributed across the 24-h day, the best cosine function was fitted according to the following formula: $(t)\hspace{0.17em}=\hspace{0.17em}M\hspace{0.17em}+\hspace{0.17em}Acos (2\uppi t/\mathrm{\rm T}\hspace{0.17em}+\hspace{0.17em}\upphi )\hspace{0.17em}+\hspace{0.17em}e(t)$. In this formula, M is the mesor, A is the amplitude, ϕ is the acrophase, Τ is the period, and e(t) is the error term. For each day the accelerometer was worn, a cosine function was fitted to determine the diurnal rest-activity parameters. Afterwards, these values were averaged across all available accelerometer days. **B** The dichotomy index was calculated as the percentage of in-bed (I) activity counts, expressed per 1-min interval, that were less than the median of out-of-bed (O) activity counts. The dichotomy index was calculated for each day the accelerometer was worn, and these values were averaged across all available days. **C** Overlap is shown between two different fictitious days the accelerometer was worn. The 24-h autocorrelation coefficient describes the average correlation between activity counts in 1-min intervals separated by 24 h. For the 24-h autocorrelation coefficient only one value was calculated, as the autocorrelation across all available accelerometer days. In this example, only day 1 and 2 are shown; however, activity counts separated by 24 h between day 2 and day 3, day 3 and 4 etc., are also included in the calculation

RAR parameters included mesor, amplitude, acrophase, circadian quotient, dichotomy index, and 24-h autocorrelation. These measures were determined based on the activity counts at all post-treatment time points. These parameters were calculated using a custom-made MATLAB program (Maastricht Instruments B.V., Maastricht, The Netherlands), and are described in more detail below. Additionally, a schematic overview of these parameters is shown in Fig. 2. Each of the parameters, except for the 24-h autocorrelation, was calculated for every valid day, and afterwards averaged over all valid days available at each post-treatment time point. For the 24-h autocorrelation, one value for each post-treatment time point was obtained based on all days the accelerometer was worn at that specific time point. For obtaining the mesor, amplitude, and acrophase, the cosinor method, widely used within RAR studies, was used [25, 26].

#### Mesor

The midline estimating statistic of the rhythm (mesor) is the 24-h rhythm-adjusted mean of the activity counts. Higher values for the mesor are indicative of increased activity over the 24-h day, whereas lower values indicate less activity [27].

#### Amplitude

The amplitude is the difference between the highest activity point of the cosinor curve (peak) and the mesor in activity counts. Higher values for amplitude indicate a larger contrast between average activity levels and peak activity [27].

#### Acrophase

The acrophase describes the clock time of the cosine weighted peak, and is expressed in decimal hours. Acrophase is often an appropriate estimate of the timing of a person’s 24h rhythm as it describes the timing of the cosine weighted peak [27].

#### Circadian quotient

The circadian quotient is calculated by dividing the amplitude by the mesor. The benefit of this measure is that, unlike unadjusted amplitude, it is correcting for average differences in activity. Higher values, i.e. higher amplitude relative to mesor, reflect a stronger RAR [16].

#### Dichotomy index

The dichotomy index (I < O) describes the percentage of in-bed (I) activity counts that are less than the median of out-of-bed (O) activity counts [28]. In this regard, wake and bed times were reported by participants in a structured 7-day sleep and dietary record. Lower values for I < O signify weaker RAR, whereas higher I < O values signal stronger RAR [28]. The I < O was calculated for each 24-h period ranging from 00:00 – 23:59h, on all valid wear days [28].

#### Twenty-four hour autocorrelation

The 24-h autocorrelation coefficient quantifies the regularity or consistency of the RAR from one day to the next, in other words between sequential days. More specifically, this coefficient describes the average correlation between activity (count) levels in 1-min epochs separated by 24 h. Higher and positive values for the 24-h autocorrelation coefficient indicate a more consistent RAR [16]. To have a representative number based on the full 7-day week at each post-treatment time point, the 24-h autocorrelation coefficient was only calculated in case of seven valid wear days. Due to non-wear on one or more days, 34% of accelerometer measurements were excluded for this parameter.

### Patient-reported outcomes

#### Fatigue

The Checklist Individual Strength (CIS), a validated 20-item questionnaire, was used to acquire a multidimensional assessment of fatigue at all post-treatment time points [29]. The CIS consists of four subscales and a total score (ranging from 20 to 140) derived from summing the individual subscales. The subscales include subjective fatigue (ranging from 8 to 56), motivation- (ranging from 4 to 28), concentration- (ranging from 5 to 25), and activity-related fatigue (ranging from 3 to 21) [30]. Higher scores on all these scales indicate more fatigue. Participants’ fatigue was also measured using the fatigue symptom scale of the European Organization for the Research and Treatment of Cancer Quality of Life Questionnaire (EORTC QLQ-C30) [31]. The fatigue symptom scale consists of three items, and based on these items, a fatigue score was calculated which ranged from 0 to 100, with higher scores indicating more fatigue. The EORTC QLQ-C30 fatigue subscale is cancer-specific and has been validated in cancer populations [31].

#### Insomnia

The validated EORTC QLQ-C30 insomnia scale was used to assess the severity of sleep problems of participants at all post-treatment time points [31]. The insomnia scale consists of one item; “Have you had trouble sleeping in the past week?” with four response options from 1 “not at all” to 4 “very much”, based on which a score was calculated ranging from 0 to 100. Higher values indicate more sleep problems (insomnia). Recently, the one-item insomnia scale of the EORTC QLQ-C30 was found to be suitable for detecting sleep problems among patients with cancer and for testing associations of those sleep problems with other variables [32]. Additionally, sleep duration in hours was determined using self-reported wake and bed times by participants in a structured 7-day sleep and dietary record which was completed at all post-treatment time points on the same days as the accelerometer was worn. If missing, sleeping times were determined based on visual inspection of acceleration data (MOX activity monitor). Based on the reported sleeping times, the midpoint of sleep was calculated for every night of each participant. The average midpoint of sleep for each post-treatment time point was determined by averaging the midpoint of sleep across all available nights of every participant at that post-treatment time point.

#### Health-related quality of life

The widely used and well-validated EORTC QLQ-C30 was used to measure cancer-specific health-related quality of life (HRQoL) at all post-treatment time points [31]. The EORTC QLQ-C30 contains a global health/QoL scale, as well as subscales for physical, role, cognitive, emotional, and social functioning [31]. All scores were converted to a 0 to 100 scale, where higher scores indicated better QoL and functioning. In the current analyses, we only included global QoL and the physical functioning scale because these were hypothesized to be most likely related to RAR [14, 20].

### Lifestyle, clinical, sociodemographic, and psychosocial factors

Clinical information, including cancer stage (I, II, III), type of treatment (including surgery, chemotherapy and/or radiotherapy), and tumor site (colon vs. rectum), were collected from medical records. Furthermore, self-reported data was retrieved on age, sex, educational level, employment status, smoking status, number of co-morbidities [33], and presence of a stoma. Trained dietitians performed anthropometric measurements at every post-treatment time point during home visits according to standardized procedures [34]. From October 2020 onwards, due to the COVID-19 pandemic, anthropometric data were self-reported. Participants’ body height and weight were measured in duplicate and then averaged to determine body mass index (BMI) in kg/m^2^. BMI was categorized using the World Health Organization (WHO) guidelines into underweight (BMI < 18.5 kg/m^2^), normal weight (18.5 ≤ BMI < 25 kg/m^2^), overweight (25 ≤ BMI < 30 kg/m^2^), or obesity (BMI ≥ 30 kg/m^2^). Dietary intake of participants, including alcohol consumption, was measured through 7-day food diaries collected at each post-treatment time point. Further, total time spent in total physical activity (all activities with an energy expenditure > 1.5 METs) was objectively determined at each post-treatment time point using the validated tri-axial MOX activity monitor. Similarly, prolonged sedentary behavior (both hours/day), i.e., time accumulated in sedentary bouts with a duration of at least 30 min, was determined at each post-treatment time point, as previously described in more detail [35]. Finally, the Hospital Anxiety and Depression Scale (HADS) was used to assess symptoms related to depression and anxiety at each post-treatment time point [36].

### Statistical analyses

Descriptive analyses summarized characteristics of the study population, encompassing RAR parameters, fatigue, insomnia, HRQoL, as well as sociodemographic, psychosocial, lifestyle, and clinical variables at each post-treatment time point. Normally distributed quantitative variables were presented as mean (± SD), whereas non-normally distributed quantitative variables were presented as median (inter-quartile range, IQR). Categorical variables were described as frequency (percentage) across classes. For describing longitudinal changes over time in RAR parameters, fatigue, insomnia, and HRQoL, time was modeled as a categorical variable in linear mixed regression models, represented by dummy variables with the measurement at 6 months post-treatment selected as reference category (based on the observed pattern of changes over time). Furthermore, Pearson’s correlation coefficients were calculated to determine the correlation between RAR parameters, total physical activity, and prolonged sedentary time.

Longitudinal analyses were performed using linear mixed models, to analyze associations of RAR parameters with fatigue, insomnia, and HRQoL from 6 weeks to 60 months post-treatment. For comparability of results across parameters, all RAR parameters were standardized by dividing individual values by the mean standard deviation across all five post-treatment time points for each RAR parameter. Mesor, amplitude, circadian quotient, dichotomy index, and 24-h autocorrelation were modeled continuously. Acrophase was modeled categorically based on tertiles, as for this variable the linearity assumption was unrealistic. Longitudinal associations were adjusted for a priori-defined confounders, based on literature and causal reasoning. Fixed (time-invariant) confounders included age at enrollment (years), sex, neo-adjuvant radio- and/or chemotherapy (yes, no), adjuvant chemotherapy (yes, no), and education level (low, medium, high). Time-variant confounders, which were measured at all post-treatment time points, included number of comorbidities (0, 1, ≥ 2), BMI (kg/m^2^), stoma (yes, no), smoking (current, former, never), employment (employed, unemployed/retired), alcohol intake (g/day), and time since diagnosis (months). Associations for acrophase were additionally adjusted for the time-variant variable midpoint of sleep to account for potential differences in the timing of sleep. The use of random slopes was tested for all models with a likelihood-ratio test. These were added when the model improved statistically significantly. In addition, inter- and intra-individual associations were disaggregated by adding centered person-mean values to the model to estimate inter-individual associations (i.e., average differences between participants over time) and individual deviations from the person-mean value to estimate intra-individual associations (i.e., within-participant changes over time) [37].

To assess potential interaction between RAR parameters and sex, chemotherapy (yes/no), BMI (categorical at 6 weeks post-treatment), and time since diagnosis (months), in relation to fatigue, insomnia, and HRQoL, interaction terms were added into the linear mixed models. Statistical significance for the interaction term was set at *P* < 0.05.

As a sensitivity analysis, to obtain more insight into the possible direction of longitudinal associations, time-lag models were used, in which RAR parameters at earlier time points were coupled with fatigue, insomnia, and HRQoL outcomes at subsequent time points to mimic a more natural direction of associations. Two additional sensitivity analyses including additional adjustment for covariates were performed. First, we investigated associations of RAR with fatigue, insomnia, and HRQoL with additional adjustment for total physical activity and prolonged sedentary time. This was done to determine if observed associations could be (partly) attributed to a more (or less) active lifestyle in general, rather than 24h rhythms of activity and rest. Second, analyses were additionally adjusted for the total anxiety and depression score of participants at 6 weeks post-treatment, to investigate potential confounding effects. Finally, with regards to acrophase, we additionally investigated the phase difference as an exposure variable, as this represents an alternative method to look at RAR timing in relation to sleep timing [38]. The phase difference was calculated as the difference in clock-hours between the acrophase and the midpoint of sleep. All statistical analyses were performed using Stata 16.0 (StataCorp LLC) with statistical significance set at *P* < 0.05 (two-sided).

## Results

Characteristics of the included study participants (*n* = 289) at 6 weeks post-treatment are presented in Table 1. About two-thirds of participants were men (67%), with an average age of 67 years (SD = 9) at enrollment. The majority of participants were colon cancer survivors (63%), whereas 37% were rectosigmoid or rectum cancer survivors. Approximately half of the participants were diagnosed with stage III CRC (44%), followed by stage I (31%), and stage II (25%). With regards to treatment, 89% of participants had surgery, 25% received radiotherapy (all except for one pre-operative), and 38% of participants received chemotherapy (18% of participants pre-operative and 29% post-operative, some received both). The average BMI of our population at 6 weeks post-treatment was 28 kg/m^2^ (SD = 5), and most participants (44%) had at least two co-morbidities. Median daily time spent in total physical activity (all activities with an energy expenditure > 1.5 METs) at 6 weeks post-treatment was 1.4 h (IQR: 1.0 – 1.9), whereas participants’ median daily time spent in prolonged sedentary behavior (in bouts with duration ≥ 30 min) was 4.8 h (IQR: 3.2 – 6.7) at 6 weeks post-treatment.

**Table 1:** Sociodemographic, lifestyle, and clinical characteristics of participants included in the Energy for Life after ColoRectal cancer (EnCoRe) study at 6 weeks after treatment

**Characteristics**	**Total, *n* = 289**^**a**^
**Age (years), mean ± SD**	67.1 ± 9.0
**Sex (male), *n* (%)**	194 (67.1)
**Education**^**b**^**, *n* (%)**	
Low	80 (27.7)
Medium	110 (38.1)
High	99 (34.3)
**BMI (kg/m**^**2**^**), mean ± SD**	27.5 ± 4.5
**BMI categorical, *n* (%)**	
Underweight: < 18.5 kg/m^2^	2 (0.7)
Healthy weight: 18.5 – 24.9 kg/m^2^	91 (31.5)
Overweight: 25.0 – 29.9 kg/m^2^	123 (42.6)
Obese: ≥ 30 kg/m^2^	73 (25.3)
**Smoking status, *n* (%)**	
Never	86 (29.8)
Former	178 (61.6)
Current	25 (8.7)
**Alcohol intake (g/day), median (IQR)**	6.2 (0.0 – 18.7)
**Employed (yes), *n* (%)**	88 (30.4)
**Stoma (yes), *n* (%)**	80 (27.7)
**Treatment, *n* (%)**	
No treatment given	13 (4.5)
Surgery alone	146 (50.5)
Radiotherapy alone	1 (0.3)
Surgery and radiotherapy	18 (6.2)
Surgery and chemotherapy	58 (20.1)
Radiotherapy and chemotherapy	18 (6.2)
Surgery, radiotherapy and chemotherapy	35 (12.1)
**Cancer type, *n* (%)**	
Colon	183 (63.3)
Rectosigmoid and rectum	106 (36.7)
**Tumour stage, *n* (%)**	
Stage I	91 (31.5)
Stage II	72 (24.9)
Stage III	126 (43.6)
**Comorbidities, *n* (%)**	
0 comorbidities	60 (20.8)
1 comorbidity	78 (27.0)
≥ 2 comorbidities	151 (52.2)
**Total physical activity (h/day), median (IQR)**	1.4 (1.0 – 1.9)
**Prolonged sedentary time**^**c**^** (h/day), median (IQR)**	4.8 (3.2 – 6.7)

*Abbreviations: BMI *Body mass index, *SD *Standard deviation, *IQR *Interquartile range

^a^Percentages may not add up to 100 due to rounding

^b^Highest attained education, low: no education, primary education, or basic vocational education; medium: advanced vocational education or senior secondary vocational education; high: senior secondary general education, higher professional education, or academic higher education

^c^Time accumulated in sedentary bouts with a duration of at least 30 min, as measured by the MOX accelerometer

### Descriptives of diurnal rest-activity rhythms, fatigue, insomnia, and HRQoL up to 60 months post CRC treatment

Descriptives of RAR parameters and fatigue, insomnia, and HRQoL from 6 weeks to 60 months post-treatment are presented in Table 2. At 6 weeks post-treatment, mesor, with higher values reflecting more activity during the 24-h day, was on average 3.71 (SD = 0.17). Similarly, amplitude, with higher values expressing a larger difference between peak- and mean activity levels, was 0.57 (SD = 0.18). Acrophase, indicating the clock-time of the peak activity, was 14:12h (IQR: 13:24h – 14:58h). Moreover, circadian quotient, which is the peak activity adjusted for mean activity with a higher value indicating a larger difference between peak and mean activity as the amplitude divided by the mesor, was 0.15 (SD = 0.04). Dichotomy index, with higher values reflecting a larger difference between daytime and nighttime activity, was 0.78 (SD = 0.21). Lastly, 24-h autocorrelation, with higher values indicating a more consistent rest-activity rhythm from one day to the next, was 0.16 (SD = 0.10). Linear mixed models showed that all RAR parameters, except for 24-h autocorrelation, increased statistically significantly from 6 weeks to 6 months post-treatment. The average absolute change in this time period was 0.03 (SD = 0.14), 0.03 (SD = 0.15), 0:01h (IQR: -0:26h – 0:39h), 0.01 (SD = 0.04), and 0.10 (SD = 0.23), for mesor, amplitude, acrophase, circadian quotient, and dichotomy index, respectively. Afterwards, from 6 to 60 months post-treatment, RAR parameters remained relatively stable, except for mesor where an increase at 60 months post-treatment was observed (0.05 (SD = 0.16)) (Table 2).

**Table 2:** Descriptive analyses of diurnal rest-activity rhythm parameters and fatigue, insomnia, and health-related quality of life in the study population of survivors of colorectal cancer from 6 weeks to 60 months post-treatment

**Characteristics**	**6 weeks post-treatment** **(*N* = 289)**	**6 months post-treatment** **(*N* = 271)**^**a**^	**12 months post-treatment** **(*N* = 229)**^**a**^	**24 months post-treatment** **(*N* = 172)**^**a**^	**60 months post-treatment** **(*N* = 87)**^**a**^
**Diurnal rest-activity parameters (mean ± SD)/(median—IQR)**					
Mesor	3.71 ± 0.17*	3.75 ± 0.17	3.75 ± 0.17	3.75 ± 0.17	3.81 ± 0.19*
Amplitude	0.57 ± 0.18*	0.61 ± 0.18	0.62 ± 0.18	0.62 ± 0.18	0.60 ± 0.19*
Acrophase (clock hours)	14:12 (13:24 – 14:58)*	14:18 (13:41 – 14:57)	14:24 (13:41 – 14:57)	14:18 (13:32 – 14:57)	14:22 (13:47 – 14:52)
Circadian quotient	0.15 ± 0.04*	0.16 ± 0.04	0.16 ± 0.04	0.16 ± 0.04	0.15 ± 0.04*
Dichotomy index	0.78 ± 0.21*	0.87 ± 0.08	0.88 ± 0.08	0.87 ± 0.09	0.88 ± 0.10
24-h autocorrelation^**b**^	0.16 ± 0.10	0.17 ± 0.09	0.17 ± 0.10	0.18 ± 0.12	0.19 ± 0.12
**EORTC QLQ-C30—range 0 – 100**^**c**^ **(mean ± SD)**					
Fatigue	28.0 ± 22.4*	21.5 ± 19.9	20.3 ± 21.5	18.5 ± 20.9*	21.3 ± 22.4
Insomnia	24.1 ± 28.3*	19.1 ± 26.8	18.6 ± 26.5	18.6 ± 26.0	16.1 ± 23.2
Physical functioning	78.0 ± 18.9*	83.5 ± 17.2	84.2 ± 18.0	85.1 ± 17.2	84.8 ± 18.3
Global QoL	75.1 ± 18.2*	78.1 ± 16.8	78.5 ± 16.6	78.8 ± 18.0	78.4 ± 16.7
**Checklist Individual Strength (CIS)**^**c**^					
Total fatigue (range 20 – 140)	62.5 ± 26.4*	56.3 ± 26.3	53.4 ± 25.7*	53.6 ± 25.9	57.1 ± 26.1
Subjective fatigue (range 8 – 56)	26.4 ± 13.0*	23.6 ± 12.5	22.2 ± 12.2*	22.0 ± 12.2	24.2 ± 12.6
Activity-related fatigue (range 3–21)	10.6 ± 5.2*	9.0 ± 5.1	8.6 ± 5.0	8.3 ± 5.1*	9.0 ± 5.5

*Abbreviations*: *SD* standard deviation, *IQR* interquartile range, *EORTC QLQ-C30* European Organization for the Research and Treatment of Cancer Quality of Life Questionnaire, *QoL* quality of life

In total, 1092 accelerometer measurements were performed between time points from 6 weeks up to 60 months post-treatment. Out of these 1092, 44 (4.0%) were excluded due to non-wear resulting in less than four valid days. For 69% of the accelerometer measurements, all seven days were available without any non-wear. For 92% of accelerometer measurements, at least six valid days were available to calculate diurnal rest-activity rhythm parameters

^a^Response rates for the follow-up time points up to 24-months were all above 90% and for 60 months 63%. The decreasing absolute numbers are largely due to the fact that participants had not yet reached all post-treatment follow-up time points at the time of data acquisition in July 2018 for measurements at 6 weeks to 24 months post-treatment and in October 2021 for 60 months post-treatment

^b^To have a representative number based on the full 7-day week, the 24-h autocorrelation coefficient was only calculated in case of seven valid wear days. Therefore, there were fewer accelerometer measurements available for this parameter. The N was 200, 183, 156, 130, and 49 for 6 weeks to 60 months post-treatment measurements respectively

^c^Higher scores on the EORTC QLQ-C30 for fatigue and insomnia, and Checklist Individual Strength (CIS) reflect more symptoms (i.e., worse fatigue or sleeping problems). Higher scores on the EORTC QLQ-C30 for physical functioning or global QoL indicate better physical functioning or a higher global QoL

^*^Significant longitudinal changes over time were observed compared to the measurement at 6 months-post treatment (linear mixed models with 6 months post-treatment as reference category, *P* < 0.05)

All fatigue and insomnia scores significantly decreased, whereas physical functioning and global QoL significantly increased from 6 weeks to 6 months post-treatment. The decline in fatigue and insomnia scores, and the increase in physical functioning and global QoL appeared to continue up until 24 months post-treatment, although these changes were mostly non-significant (Table 2). Generally, moderate to strong positive correlations were observed among RAR parameters and with total physical activity, whereas moderate negative correlations were observed with sedentary behavior (Supplementary Fig. 1).

### Longitudinal associations of diurnal rest-activity rhythm parameters with fatigue, insomnia, and HRQoL

Table 3 shows confounder-adjusted longitudinal associations of RAR parameters mesor, amplitude, acrophase, circadian quotient, dichotomy index, and 24-h autocorrelation with fatigue, insomnia, and HRQoL from 6 weeks to 60 months post-treatment, including overall, intra- and inter-individual associations.

**Table 3:** Longitudinal associations of diurnal rest-activity rhythm parameters with fatigue, insomnia, and health-related quality of life in the study population of survivors of colorectal cancer between 6 weeks and 60 months post-treatment

		**Checklist Individual Strength (CIS)**			**EORTC-QLQ-C30**			
**Diurnal rest-activity rhythm parameters per SD**		Total fatigue (20 – 140)	Subjective Fatigue (8 – 56)	Activity Fatigue (3 – 21)	Fatigue (0 – 100)	Insomnia (0 – 100)	Global QoL (0 – 100)	Physical Functioning (0 – 100)
		*Β*^*a*^ (95% CI)	*β* (95% CI)	*β* (95% CI)	*β* (95% CI)	*β* (95% CI)	*β* (95% CI)	*β* (95% CI)
**Mesor** *(reflects mean activity level)*	Adjusted^b,c^	**-5.3 (-6.9, -3.6)**	**-2.2 (-3.0, -1.4)**	-**1.5 (-1.8, -1.1)**	**-3.4 (-5.0, -1.8)**	-2.0 (-4.0, 0.0)	**3.7 (2.5, 4.9)**	**3.2 (2.1, 4.4)**
	Intra^d^	**-4.8 (-6.8, -2.8)**	**-2.0 (-3.0, -1.0)**	**-1.3 (-1.8, -0.9)**	**-3.8 (-5.7, -1.9)**	**-3.2 (-6.0, -0.5)**	**3.5 (1.9, 5.0)**	**2.3 (1.0, 3.5)**
	Inter^e^	**-6.3 (-9.1, -3.4)**	**-2.6 (-4.0, -1.3)**	**-1.7 (-2.3, -1.2)**	**-3.2 (-5.5, -0.9)**	-0.7 (-3.5, 2.1)	**4.5 (2.7, 6.3)**	**6.5 (4.7, 8.3)**
**Amplitude** *(reflects difference between peak- and mean activity levels)*	Adjusted^b,c^	**-5.5 (-7.1, -3.9)**	**-2.5 (-3.3, -1.6)**	**-1.3 (-1.7, -1.0)**	**-4.7 (-6.2, -3.1)**	**-2.4 (-4.3, -0.4)**	**4.1 (3.0, 5.2)**	**4.7 (3.7, 5.7)**
	Intra^d^	**-4.9 (-6.8, -3.0)**	**-2.3 (-3.2, -1.3)**	**-1.2 (-1.6, -0.7)**	**-5.4 (-7.2, -3.6)**	**-2.9 (-5.5, -0.2)**	**3.7 (2.2, 5.2)**	**3.7 (2.5, 4.9)**
	Inter^e^	**-6.8 (-9.7, -3.9)**	**-3.0 (-4.4, -1.7)**	**-1.6 (-2.2, -1.1)**	**-4.3 (-6.6, -2.1)**	-1.9 (-4.7, 0.9)	**5.4 (3.7, 7.2)**	**8.3 (6.5, 10.0)**
**Circadian Quotient** *(reflects peak activity adjusted for mean activity levels)*	Adjusted^b,c^	**-5.1 (-6.6, -3.6)**	**-2.3 (-3.1, -1.5)**	**-1.2 (-1.5, -0.9)**	**-4.5 (-6.0, -3.0)**	**-2.3 (-4.2, -0.4)**	**3.9 (2.7, 5.0)**	**4.6 (3.6, 5.6)**
	Intra^d^	**-4.5 (-6.3, -2.7)**	**-2.1 (-3.0, -1.2)**	**-1.0 (-1.4, -0.6)**	**-5.1 (-6.8, -3.4)**	-2.5 (-5.0, 0.1)	**3.4 (2.0, 4.8)**	**3.5 (2.4, 4.6)**
	Inter^e^	**-6.6 (-9.4, -3.8)**	**-3.0 (-4.3, -1.7)**	**-1.6 (-2.1, -1.0)**	**-4.3 (-6.6, -2.1)**	-2.1 (-4.9, 0.7)	**5.4 (3.6, 7.1)**	**8.3 (6.5, 10.0)**
**Dichotomy Index** *(reflects difference between daytime and nighttime activity)*	Adjusted^b,c^	**-3.1 (-4.2, -2.1)**	**-1.0 (-1.4, -0.5)**	**-1.0 (-1.2, -0.7)**	**-2.9 (-4.0, -1.8)**	-1.0 (-2.2, 0.3)	**1.7 (0.7, 2.6)**	**2.3 (1.5, 3.1)**
	Intra^d^	**-2.5 (-3.5, -1.4)**	**-1.1 (-1.6, -0.5)**	**-0.7 (-0.9, -0.4)**	**-2.0 (-3.0, -1.0)**	-1.1 (-2.6, 0.4)	0.7 (-0.2, 1.5)	**1.0 (0.3, 1.7)**
	Inter^e^	-1.8 (-4.2, 0.5)	-0.5 (-1.6, 0.6)	**-0.8 (-1.3, -0.4)**	-1.0 (-2.9, 0.8)	-0.6 (-3.0, 1.7)	1.1 (-0.4, 2.7)	**3.9 (2.4, 5.4)**
**24-h autocorrelation coefficient** *(rhythm consistency across days)*	Adjusted^b,c^	-1.1 (-2.9, 0.7)	**-1.3 (-2.1, -0.4)**	-0.1 (-0.4, 0.3)	**-3.0 (-4.6, -1.4)**	-1.2 (-3.4, 1.1)	**1.5 (0.1, 2.9)**	**4.1 (2.9, 5.4)**
	Intra^d^	0.3 (-1.8, 2.4)	-0.2 (-1.3, 0.9)	0.2 (-0.3, 0.7)	-1.3 (-3.5, 0.8)	-2.0 (-5.1, 1.2)	0.1 (-1.7, 1.9)	**1.9 (0.5, 3.4)**
	Inter^e^	**-4.5 (-7.7, -1.3)**	**-2.7 (-4.2, -1.2)**	-0.4 (-1.1, 0.2)	**-4.5 (-7.0, -2.0)**	-0.4 (-3.4, 2.7)	**2.9 (0.9, 4.9)**	**5.7 (3.8, 7.7)**
**Acrophase, per tertile**^**f**^		Total fatigue (20 – 140)	Subjective Fatigue (8 – 56)	Activity Fatigue (3 – 21)	Fatigue (0 -100)	Insomnia (0–100)	Global QoL(0-100)	Physical Functioning (0-100)
		*β*^*a*^ (95% CI)	*β* (95% CI)	*β* (95% CI)	*β* (95% CI)	*β* (95% CI)	*β* (95% CI)	*β* (95% CI)
**Acrophase** *(reflects clock-time of peak activity)*	Adjusted, tertile 1	REF	REF	REF	REF	REF	REF	REF
	Adjusted, tertile 2	-0.2 (-3.2, 2.9)	0.2 (-1.4, 1.7)	-0.2 (-0.8, 0.5)	-0.5 (-3.3, 2.3)	1.1 (-2.9, 5.0)	-0.6 (-2.9, 1.7)	-1.2 (-3.1, 0.7)
	Adjusted, tertile 3	0.4 (-3.2, 4.0)	0.3 (-1.5, 2.1)	0.2 (-0.6, 1.0)	-0.7 (-3.9, 2.6)	3.0 (-1.5, 7.5)	-0.9 (-3.6, 1.8)	-1.4 (-3.7, 0.8)

Values in bold are statistically significant (*P* < 0.05)

*Abbreviations: CIS* Checklist Individual Strength, *EORTC QLQ-C30* European Organization for the Research and Treatment of Cancer Quality of Life Questionnaire

^a^The β-coefficients indicate the overall longitudinal difference in the outcome score using linear mixed models per standard deviation (SD) increase in diurnal rest-activity rhythms parameter. This SD was calculated by summing the SDs of each diurnal rest-activity rhythm parameter at each time point, and then dividing this number by 5 (number of time points). The SDs were as follows: mesor 0.175, amplitude 0.180, circadian quotient 0.042, dichotomy index 0.114, and 24-h autocorrelation coefficient 0.107

^b^Linear mixed-models adjusted for sex (male/female), age at enrollment (years), weeks since end of treatment (weeks), neo-adjuvant therapy (yes/no), adjuvant therapy (yes/no), comorbidities (0, 1, ≥ 2), BMI (kg/m^2^), stoma (yes/no), smoking (former, current, never), employment status (yes/no), and alcohol intake (g/day)

^c^A random slope was added to the model when the model improved statistically significantly using a likelihood-ratio test

^d^The β-coefficients indicate the average change in the outcome score over time when exposure increases with 1 SD between time points from 6 weeks to 60 months post-treatment within individuals

^e^The β-coefficients indicate the average difference in the outcome score between individuals differing by 1 SD in average exposure across all time points from 6 weeks to 60 months post-treatment

^f^Tertiles were calculated based on the distribution of values at each individual post-treatment time point. Ranges (clock-hours) were as follows: 6 weeks post-treatment, tertile 1 01:24 – 13:46, tertile 2 13:47 – 14:42, tertile 3 14:43 – 20:10; 6 months post-treatment, tertile 1 08:19 – 13:54, tertile 2 13:55 – 14:43, tertile 3 14:44 – 19:05; 12 months post-treatment, tertile 1 10:45 – 13:56, tertile 2 13:57 – 14:44, tertile 3 14:45 – 17:48; 24 months post-treatment, tertile 1 10:35 – 13:47, tertile 2 13:48 – 14:40, tertile 3 14:41 – 17:47; 60 months post-treatment, tertile 1 08:02 – 14:01, tertile 2 14:02 – 14:38, tertile 3 14:39 – 17:13

#### Fatigue

Higher values for mesor, amplitude, circadian quotient, and dichotomy index were statistically significantly associated with lower values for all fatigue outcomes (Table 3). For each SD increase in mesor (0.175), the CIS total score decreased on average by 5.3 points (95% CI: -6.9, -3.6), whereas each SD increase in amplitude (0.180) was associated with a decrease of 5.5 points (-7.1, -3.9) in the CIS total fatigue score. Similarly, each SD increase in circadian quotient (0.042) and dichotomy index (0.114) was significantly associated with decreases of 5.1 (-6.6, -3.6) and 3.1 points (-4.2, -2.1) in CIS total fatigue, respectively. Similar results were observed for the fatigue score as measured through the EORTC QLQ-C30 scale. Although for 24-h autocorrelation inverse associations were observed with all fatigue outcomes, only associations with subjective fatigue (CIS) (-1.3; -2.1, -0.4) and fatigue as measured by the EORTC subscale (-3.0; -4.6, -1.4) were statistically significant. No significant associations of the acrophase with fatigue were observed.

Most of the described associations with fatigue were driven by both intra-individual changes over time and inter-individual differences. However, generally, inter-individual associations were slightly stronger as compared to intra-individual associations. For example, inter-individual associations showed that participants whose average mesor was one SD (0.175) higher than other participants, reported lower levels of CIS total fatigue (β: -6.3; 95%CI: -9.1, -3.4), whereas for participants intra-individually increasing their mesor by one SD a smaller inverse association was found (-4.8; -6.8, -2.8).

#### Insomnia

Each SD increase in amplitude (0.180) and circadian quotient (0.042) was significantly associated with lower insomnia scores over time (-2.4; -4.3, -0.4 and -2.3; -4.2, -0.4, respectively). With regards to both amplitude and circadian quotient, this overall association was mainly driven by intra-individual changes over time (-2.9; -5.5, -0.2 and -2.5; -5.0, 0.1, respectively). For the RAR parameters mesor, acrophase, dichotomy index, and 24-h autocorrelation, no significant overall associations with insomnia were found (Table 3).

#### Physical functioning and global QoL

Higher values for mesor, amplitude, circadian quotient, dichotomy index, and 24-h autocorrelation were statistically significantly associated with better physical functioning and global QoL (Table 3). For each SD increase in mesor (0.175), physical functioning was on average 3.2 points (2.1, 4.4) higher and global QoL 3.7 points (2.5, 4.9) higher. Similarly, for each SD increase in amplitude (0.180), physical functioning was on average 4.7 points (3.7, 5.7) higher and global QoL 4.1 points (3.0, 5.2) higher. For each SD increase in circadian quotient (0.042), physical functioning was on average 4.6 points (3.6, 5.6) and global QoL 3.9 points (2.7, 5.0) higher. Similarly, for each SD increase in dichotomy index (0.114), physical functioning was on average 2.3 points (1.5, 3.1) higher and global QoL 1.7 points (0.7, 2.6) higher. Finally, each SD increase in 24-h autocorrelation (0.107) was associated with better physical functioning (4.1; 2.9, 5.4) and a higher global QoL (1.5; 0.1, 2.9). No significant associations of acrophase with either of these outcomes were observed.

Most of the described associations with physical functioning and global QoL appeared to be mainly driven by inter-individual differences over time, although also significant intra-individual associations were observed (Table 3). For example, inter-individual associations showed that participants whose average 24-h autocorrelation was one SD (0.107) higher than other participants, reported better physical functioning (5.7; 3.8, 7.7), whereas for participants intra-individually increasing their 24-h autocorrelation by one SD a smaller association was found (1.9; 0.5, 3.4).

### Interaction and sensitivity analyses

For some of the described overall associations, significant interaction effects were observed with time since end of treatment. Therefore, in case of significant interaction, stratified effects for each post-treatment time point are visualized in Supplementary Fig. 2. In general, observed associations of RAR parameters with fatigue, insomnia, and HRQoL were strongest at the 6 weeks post-treatment time point, whereas for the RAR variable dichotomy index associations were strongest at 2–5 years post-treatment. No significant interaction effects were found for sex, chemotherapy, and BMI.

In the sensitivity analyses additionally adjusting for total physical activity and prolonged sedentary time, we found mostly similar associations as compared to the main results (Supplementary Fig. 3). Although slightly attenuated, most associations were in a similar direction and remained statistically significant, except for the associations of acrophase with fatigue outcomes, physical functioning, and global QoL, where associations were strengthened. Specifically, participants in higher tertiles, indicating a later clock-time for the cosine weighted peak, reported on average statistically significantly higher activity-related fatigue and lower physical functioning. Results from the sensitivity analysis additionally adjusting for anxiety and depression scores of participants at 6 weeks post-treatment were comparable to the results of the main analyses (Supplementary Fig. 4). In comparison with the results of the main analyses, the overall longitudinal associations of RAR parameters with fatigue, insomnia, and HRQoL as observed in the time-lag analyses were attenuated, and many betas were not statistically significant anymore (Supplementary Fig. 5). Finally, no significant associations were observed in the sensitivity analyses were we looked at the longitudinal association between the phase difference and fatigue, insomnia, and HRQoL (Supplementary Table 1).

## Discussion

To our best knowledge, this study is the first to analyze how objectively assessed RAR are longitudinally associated with fatigue, insomnia, and HRQoL in survivors of CRC from 6 weeks up to 5 years post-treatment. In general, we observed that fatigue and insomnia decreased and HRQoL improved in survivors of CRC following treatment, suggesting recovery. However, some survivors still suffer from these complaints even after a prolonged period of time. Our main findings were that higher values for RAR parameters mesor, amplitude, circadian quotient, dichotomy index, and 24-h autocorrelation were significantly associated with lower fatigue and better physical functioning and global QoL over time. In addition, a higher amplitude and circadian quotient were significantly associated with less insomnia over time. For acrophase, no significant associations were observed with any of the outcomes. In general, the observed associations were in line with our hypothesis, highlighting that having a generally more active (mesor) and consistent (24-h autocorrelation) RAR, with a pronounced peak activity (amplitude) and a marked difference between daytime and nighttime activity (dichotomy index), was associated with less fatigue and a higher HRQoL in the five years after CRC treatment.

Our findings are in line with findings from prospective and cross-sectional studies in patients with metastatic CRC showing that a higher dichotomy index and 24-h autocorrelation coefficient were associated with lower fatigue and better physical functioning and global QoL [14, 20, 21]. Furthermore, we showed that also higher scores for other RAR parameters including mesor, amplitude, and circadian quotient were associated with lower fatigue and a better HRQoL. As far as we know, we are the first to report these associations in survivors of CRC up to 5 years post-treatment, although similar associations of mesor, amplitude, and circadian quotient with fatigue and HRQoL have been reported in breast cancer patients [15, 18, 19]. Our findings with regards to insomnia are in line with cross-sectional studies conducted in patients with breast and lung cancer, showing that a higher mesor, dichotomy index, and 24-h autocorrelation were associated with a better sleep quality, albeit not statistically significant in our study [39, 40]. In addition, we found statistically significant associations between a higher amplitude and circadian quotient and lower insomnia.

Previous research shows that survivors of CRC with more time spent in physical activity and less time spent sedentary reported on average lower levels of fatigue and a better HRQoL up to ten years post-diagnosis [11, 12, 41, 42]. This highlights that physical behavior could be an important lifestyle factor that may potentially empower patients with CRC to reduce their symptoms and subsequently improve their HRQoL. In the current study, we performed a sensitivity analysis where we additionally adjusted the associations between RAR parameters and fatigue, insomnia, and HRQoL for total physical activity and prolonged sedentary time, which showed high correlations with some of these parameters. The direction of observed associations was similar, with most associations remaining statistically significant. This finding suggests that independent of the total amount of physical activity or sedentary time itself, aspects related to RAR are also relevant for fatigue, insomnia, and HRQoL. A possible underlying mechanism that could explain the observed association may be that the timing of physical behavior could influence peripheral clocks which affect centrally (SCN) controlled circadian rhythms [9, 10]. If these circadian rhythms are disrupted or desynchronized, for example by not optimally timed physical activity, this may potentially influence fatigue, insomnia, and consequently HRQoL. In this regard, inflammation could play an important role as it is connected to both circadian rhythm disruption as well as patient-reported outcomes such as fatigue [43, 44]. However, also other common upstream influences affecting both RAR and patient-reported outcomes could play a role.

In our study, we observed significant intra-individual associations of mesor, amplitude, circadian quotient, dichotomy index, and 24-h autocorrelation with fatigue, physical functioning, and/or global QoL. These observed intra-individual associations indicate that an increase in these RAR parameters within participants after CRC treatment was significantly associated with decreased fatigue symptoms and/or increased HRQoL. Therefore, it would be interesting to investigate in an intervention study if patients with CRC with a tendency for a disrupted RAR can be assisted in restoring their RAR through targeting the relevant elements, and if this would also reduce their symptoms and increase their HRQoL. Most of our observed overall associations for the EORTC QLQ-C30 subscales appeared to be clinically relevant per 1 SD increase in RAR parameter, according to previously published thresholds to interpret observed changes [45, 46]. Largest intra-individual changes in RAR parameters were observed between 6 weeks and 6 months post-treatment, but these changes were still small compared to 1 SD. Nevertheless, the observed changes in RAR parameters still highlight the potential of interventions focusing on these parameters which may induce larger changes than we observed in our observational data. Ideally, these interventions should focus on the time-period shortly after CRC treatment, as we then observed the strongest associations (Supplementary Fig. 2).

A major strength of the current study is its prospective design with repeated and in-depth measurements of both RAR and patient-reported outcomes in a large group of survivors of CRC. Moreover, RAR of participants were objectively assessed using high-quality data of the 7-day worn tri-axial MOX activity monitor. Other strengths include the relatively high response rates at all post-treatment time points (up to 24 months > 90%, after 60 months post-treatment > 63%), small amounts of missing data as a result of intensive data collection, and the wide range of information collected on potential confounders. Finally, using linear mixed models enabled us to separate overall longitudinal associations into inter- and intra-individual associations, thereby providing valuable insights into the nature of observed relations over time. Despite the strengths of the current study, some limitations should be considered. This study is an observational study, which limits our ability to draw conclusions regarding causality, despite thorough adjustment for confounders in our analyses. Compared with results of the main analyses, overall longitudinal associations of RAR with fatigue, insomnia, and HRQoL were attenuated in the time-lag analyses, with many associations losing statistical significance. This indicates that observed associations are likely reciprocal. More specifically, RAR may have affected fatigue, insomnia, and HRQoL, but at the same time these outcomes could in turn also have altered RAR. Future randomized controlled trials should further investigate whether improving RAR can reduce fatigue and insomnia and improve HRQoL. An additional limitation could be the limited response rate at diagnosis to participate in the EnCoRe study (45%), which may have resulted in selection bias affecting the generalizability of our results. It is reasonable to think that individuals with worst RAR and most complaints did not participate, which may have led to an attenuation of our observed associations. In addition, because MOX activity monitor data was not collected before the start of treatment, we were unable to describe changes in activity habits and RAR parameters from pre-treatment to post-treatment. Finally, we cannot rule out the possibility of chance findings due to the high number of statistical tests performed. Nevertheless, the consistent significant and strength of longitudinal associations of RAR parameters with fatigue, insomnia, and HRQoL emphasize the importance of our findings.

## Conclusions

Our findings show that in the first five years after CRC treatment, adhering to a generally more active and consistent RAR, with a pronounced peak activity and a marked difference between daytime and nighttime activity was found to be associated with lower fatigue, insomnia, and a better HRQoL among survivors of CRC. These findings provide valuable insights, which could help to fuel and design future intervention studies to investigate if restoring RAR has the potential to empower fatigued survivors of CRC to reduce fatigue and insomnia complaints and improve their HRQoL.

### Supplementary Information

**Additional file 1: Supplementary Figure 1.** Figure comparing the Pearson correlation coefficients (n=1048) between diurnal rest-activity rhythms parameters, total physical activity, and prolonged sedentary time (time accumulated in sedentary bouts with a duration of at least 30 min, as measured by the MOX accelerometer) using data of all post-treatment time points. Abbreviations: CQ, circadian quotient; DI, dichotomy index; AC, 24-h autocorrelation; PA, physical activity; ST, sedentary time.

**Additional file 2: Supplementary Figure 2.** Figure illustrating significant interaction effects of overall longitudinal associations between diurnal rest-activity rhythm parameters with fatigue, insomnia, and HRQoL with time since end of treatment. Stratified effects are shown separately for each post-treatment time point, and significantly different effects as compared to 6 weeks post-treatment are indicated with an asterisk. The P-value for trend indicates if the association between RAR parameters and outcomes differs significantly per 6 months increase in time since end of treatment. In case of no interaction, a horizontal line would be expected.

**Additional file 3: Supplementary Figure 3. **Figure comparing the main results of the overall associations between diurnal rest-activity rhythms parameters and fatigue, insomnia, and HRQoL, and similar associations additionally adjusted for total physical activity and prolonged sedentary time (hours/day). Abbreviations: ME, mesor; AM, amplitude; AC2, acrophase tertile 2; AC3, acrophase tertile 3; CQ, circadian quotient; DI, dichotomy index; A24, 24-h autocorrelation.

**Additional file 4: Supplementary Figure 4. **Figure comparing the main results of the overall associations between diurnal rest-activity rhythms parameters and fatigue, insomnia, and HRQoL, and similar associations additionally adjusted for anxiety and depression levels. Abbreviations: ME, mesor; AM, amplitude; AC2, acrophase tertile 2; AC3, acrophase tertile 3; CQ, circadian quotient; DI, dichotomy index; A24, 24-h autocorrelation.

**Additional file 5: Supplementary Figure 5. **Figure comparing the main results of the overall associations between diurnal rest-activity rhythms parameters and fatigue, insomnia, and HRQoL, and similar associations from a time-lag model (exposure at one post-treatment time point combined with outcomes at the next post-treatment time point). Abbreviations: ME, mesor; AM, amplitude; AC2, acrophase tertile 2; AC3, acrophase tertile 3; CQ, circadian quotient; DI, dichotomy index; A24, 24-h autocorrelation.

**Additional file 6.**

## Data Availability

Data described in the manuscript, code book, and analytic code will be made available upon request pending (e.g., application and approval, payment, other). Requests for data of the EnCoRe study can be sent to Dr. Martijn Bours, Department of Epidemiology, GROW School for Oncology and Reproduction, Maastricht University, the Netherlands (email: m.bours@maastrichtuniversity.nl).

## Abbreviations

BMI: Body mass index
CIS: Checklist Individual Strength
CRC: Colorectal cancer
EnCoRe: Energy for Life after Colorectal Cancer
EORTC QLQ-C30: European Organization for the Research and Treatment of Cancer Quality of Life Questionnaire
HADS: Hospital Anxiety and Depression Scale
HRQoL: Health-related quality of life
IQR: Interquartile range
RAR: Diurnal rest-activity rhythm
SCN: Suprachiasmatic nucleus
SD: Standard deviation
SQUASH: Short Questionnaire to Assess Health-Enhancing Physical Activity
WCRF: World Cancer Research Fund
WHO: World Health Organization

## References

[1] Sung H, Ferlay J, Siegel RL, Laversanne M, Soerjomataram I, Jemal A (2021). Global cancer statistics 2020: GLOBOCAN estimates of incidence and mortality worldwide for 36 cancers in 185 countries. CA Cancer J Clin..

[2] Siegel RL, Miller KD, Jemal A (2018). Cancer statistics, 2018. CA Cancer J Clin..

[3] Arndt V, Merx H, Stegmaier C, Ziegler H, Brenner H (2004). Quality of life in patients with colorectal cancer 1 year after diagnosis compared with the general population: a population-based study. J Clin Oncol.

[4] O’Gorman C, Stack J, O’Ceilleachair A, Denieffe S, Gooney M, McKnight M (2018). Colorectal cancer survivors: an investigation of symptom burden and influencing factors. BMC Cancer.

[5] Howell D, Oliver T, Keller-Olaman S, Davidson J, Garland S, Samuels C (2014). Sleep disturbance in adults with cancer: a systematic review of evidence for best practices in assessment and management for clinical practice. Ann Oncol.

[6] Innominato PF, Roche VP, Palesh OG, Ulusakarya A, Spiegel D, Lévi FA (2014). The circadian timing system in clinical oncology. Ann Med.

[7] Saligan LN, Olson K, Filler K, Larkin D, Cramp F, Sriram Y (2015). The biology of cancer-related fatigue: a review of the literature. Support Care Cancer.

[8] Koronowski KB, Sassone-Corsi P (2021). Communicating clocks shape circadian homeostasis. Science..

[9] Lee Y (2021). Roles of circadian clocks in cancer pathogenesis and treatment. Exp Mol Med.

[10] Hower IM, Harper SA, Buford TW (2018). Circadian rhythms, exercise, and cardiovascular health. J Circadian Rhythms..

[11] Kenkhuis MF, Van Roekel EH, Breedveld-Peters JJ, Breukink SO, Janssen-Heijnen ML, Keulen ET (2021). Longitudinal associations of sedentary behavior and physical activity with quality of life in colorectal cancer survivors. Med Sci Sports Exerc.

[12] van Roekel EH, Duchâteau J, Bours M, Van Delden L, Breedveld-Peters J, Koole J (2020). Longitudinal associations of light-intensity physical activity with quality of life, functioning and fatigue after colorectal cancer. Qual Life Res.

[13] Van Blarigan EL, Meyerhardt JA (2015). Role of physical activity and diet after colorectal cancer diagnosis. J Clin Oncol.

[14] Mormont M-C, Waterhouse J, Bleuzen P, Giacchetti S, Jami A, Bogdan A (2000). Marked 24-h rest/activity rhythms are associated with better quality of life, better response, and longer survival in patients with metastatic colorectal cancer and good performance status. Clin Cancer Res.

[15] Berger AM, Wielgus K, Hertzog M, Fischer P, Farr L (2010). Patterns of circadian activity rhythms and their relationships with fatigue and anxiety/depression in women treated with breast cancer adjuvant chemotherapy. Support Care Cancer.

[16] Berger AM, Hertzog M, Geary CR, Fischer P, Farr L (2012). Circadian rhythms, symptoms, physical functioning, and body mass index in breast cancer survivors. J Cancer Surviv.

[17] Roveda E, Bruno E, Galasso L, Mulè A, Castelli L, Villarini A (2019). Rest-activity circadian rhythm in breast cancer survivors at 5 years after the primary diagnosis. Chronobiol Int.

[18] Sultan A, Choudhary V, Parganiha A (2017). Worsening of rest-activity circadian rhythm and quality of life in female breast cancer patients along progression of chemotherapy cycles. Chronobiol Int.

[19] Liu L, Rissling M, Neikrug A, Fiorentino L, Natarajan L, Faierman M (2013). Fatigue and circadian activity rhythms in breast cancer patients before and after chemotherapy: a controlled study. Fatigue..

[20] Innominato PF, Focan C, Gorlia T, Moreau T, Garufi C, Waterhouse J (2009). Circadian rhythm in rest and activity: a biological correlate of quality of life and a predictor of survival in patients with metastatic colorectal cancer. Can Res.

[21] Innominato PF, Komarzynski S, Palesh OG, Dallmann R, Bjarnason GA, Giacchetti S (2018). Circadian rest-activity rhythm as an objective biomarker of patient-reported outcomes in patients with advanced cancer. Cancer Med.

[22] van Roekel EH, Bours MJ, de Brouwer CP, Ten Napel H, Sanduleanu S, Beets GL (2014). The applicability of the international classification of functioning, disability, and health to study lifestyle and quality of life of colorectal cancer survivors. Cancer Epidemiol Biomark Prev.

[23] Annegarn J, Spruit MA, Uszko-Lencer NH, Vanbelle S, Savelberg HH, Schols AM (2011). Objective physical activity assessment in patients with chronic organ failure: a validation study of a new single-unit activity monitor. Arch Phys Med Rehabil..

[24] Bijnens W, Aarts J, Stevens A, Ummels D, Meijer K (2019). Optimization and validation of an adjustable activity classification algorithm for assessment of physical behavior in elderly. Sensors.

[25] Nelson W (1979). Methods for cosinor-rhythmometry. Chronobiologia.

[26] Halberg F, Katinas GS (1973). Chronobiologic glossary of the International Society for the Study of Biologic Rhythms. Int J Chronobiol.

[27] Lentz MJ (1990). Time-series analysis-cosinor analysis: a special case. West J Nurs Res.

[28] Natale V, Innominato PF, Boreggiani M, Tonetti L, Filardi M, Parganiha A (2015). The difference between in bed and out of bed activity as a behavioral marker of cancer patients: A comparative actigraphic study. Chronobiol Int.

[29] Vercoulen JH, Swanink CM, Fennis JF, Galama JM, van der Meer JW, Bleijenberg G (1994). Dimensional assessment of chronic fatigue syndrome. J Psychosom Res.

[30] Servaes P, van der Werf S, Prins J, Verhagen S, Bleijenberg G (2001). Fatigue in disease-free cancer patients compared with fatigue in patients with chronic fatigue syndrome. Support Care Cancer.

[31] Aaronson NK, Ahmedzai S, Bergman B, Bullinger M, Cull A, Duez NJ (1993). The European Organization for Research and Treatment of Cancer QLQ-C30: a quality-of-life instrument for use in international clinical trials in oncology. J Natl Cancer Inst.

[32] Hofmeister D, Schulte T, Hinz A (2020). Sleep problems in cancer patients: a comparison between the Jenkins Sleep Scale and the single-item sleep scale of the EORTC QLQ-C30. Sleep Med.

[33] Sangha O, Stucki G, Liang MH, Fossel AH, Katz JN (2003). The Self-Administered Comorbidity Questionnaire: a new method to assess comorbidity for clinical and health services research. Arthritis Rheum.

[34] Kenkhuis M-F, Van Roekel EH, Koole JL, Breedveld-Peters JJ, Breukink SO, Janssen-Heijnen ML (2021). Increases in adipose tissue and muscle function are longitudinally associated with better quality of life in colorectal cancer survivors. Sci Rep.

[35] Van Roekel EH, Winkler EA, Bours MJ, Lynch BM, Willems PJ, Meijer K (2016). Associations of sedentary time and patterns of sedentary time accumulation with health-related quality of life in colorectal cancer survivors. Prev Med Rep.

[36] Zigmond AS, Snaith RP (1983). The hospital anxiety and depression scale. Acta Psychiatr Scand.

[37] Twisk JW, de Vente W (2019). Hybrid models were found to be very elegant to disentangle longitudinal within-and between-subject relationships. J Clin Epidemiol.

[38] Stone JE, Aubert XL, Maass H, Phillips AJ, Magee M, Howard ME (2019). Application of a limit-cycle oscillator model for prediction of circadian phase in rotating night shift workers. Sci Rep.

[39] Berger AM, Farr LA, Kuhn BR, Fischer P, Agrawal S (2007). Values of sleep/wake, activity/rest, circadian rhythms, and fatigue prior to adjuvant breast cancer chemotherapy. J Pain Symptom Manage.

[40] Chen H-M, Wu Y-C, Tsai C-M, Tzeng J-I, Lin C-C (2015). Relationships of circadian rhythms and physical activity with objective sleep parameters in lung cancer patients. Cancer Nurs.

[41] Lynch BM, van Roekel EH, Vallance JK (2016). Physical activity and quality of life after colorectal cancer: overview of evidence and future directions. Expert Review of Quality of Life in Cancer Care.

[42] Eyl RE, Xie K, Koch-Gallenkamp L, Brenner H, Arndt V (2018). Quality of life and physical activity in long-term (≥ 5 years post-diagnosis) colorectal cancer survivors-systematic review. Health Qual Life Outcomes.

[43] Wright KP, Drake AL, Frey DJ, Fleshner M, Desouza CA, Gronfier C (2015). Influence of sleep deprivation and circadian misalignment on cortisol, inflammatory markers, and cytokine balance. Brain Behav Immun.

[44] Bower JE, Lamkin DM (2013). Inflammation and cancer-related fatigue: mechanisms, contributing factors, and treatment implications. Brain Behav Immun.

[45] Cocks K, King M, Velikova G, de Castro JG, St-James MM, Fayers P (2012). Evidence-based guidelines for interpreting change scores for the European Organisation for the Research and Treatment of Cancer Quality of Life Questionnaire Core 30. Eur J Cancer.

[46] Revicki D, Hays RD, Cella D, Sloan J (2008). Recommended methods for determining responsiveness and minimally important differences for patient-reported outcomes. J Clin Epidemiol.

