# Supplementary material for: Longitudinal associations of diurnal rest-activity rhythms with fatigue, insomnia, and health-related quality of life in survivors of colorectal cancer up to 5 years post-treatment
Source: Int J Behav Nutr Phys Act. 2024 May 2;21:51. doi: 10.1186/s12966-024-01601-x (PMC11067118; doi:10.1186/s12966-024-01601-x)
Supplement: Supplementary file 1 — Additional file 1: Supplementary Figure 1. Figure comparing the Pearson correlation coefficients (n=1048) between diurnal rest-activity rhythms parameters, total physical activity, and prolonged sedentary time (time accumulated in sedentary bouts with a duration of at least 30 min, as measured by the MOX accelerometer) using data of all post-treatment time points. Abbreviations: CQ, circadian quotient; DI, dichotomy index; AC, 24-h autocorrelation; PA, physical activity; ST, sedentary time. [file 12966_2024_1601_MOESM1_ESM.pdf]

	Mesor	Amplitude	Acrophase	CQ	DI	24-h AC	Total PA	Prolonged ST
**Mesor**	1							
**Amplitude**	0,755	1						
**Acrophase**	0,096	0,021	1					
**CQ**	0,676	0,992	0,008	1				
**DI**	0,486	0,475	0,627	0,459	1			
**24-h AC**	0,339	0,512	-0,058	0,517	0,195	1		
**Total PA**	0,833	0,806	0,222	0,753	0,597	0,478	1	
**Prolonged ST**	-0,645	-0,549	-0,437	-0,513	-0,716	-0,148	-0,621	1
