# Supplementary material for: Longitudinal associations of diurnal rest-activity rhythms with fatigue, insomnia, and health-related quality of life in survivors of colorectal cancer up to 5 years post-treatment
Source: Int J Behav Nutr Phys Act. 2024 May 2;21:51. doi: 10.1186/s12966-024-01601-x (PMC11067118; doi:10.1186/s12966-024-01601-x)

A) CIS – Total Fatigue (Scale 20 – 140)

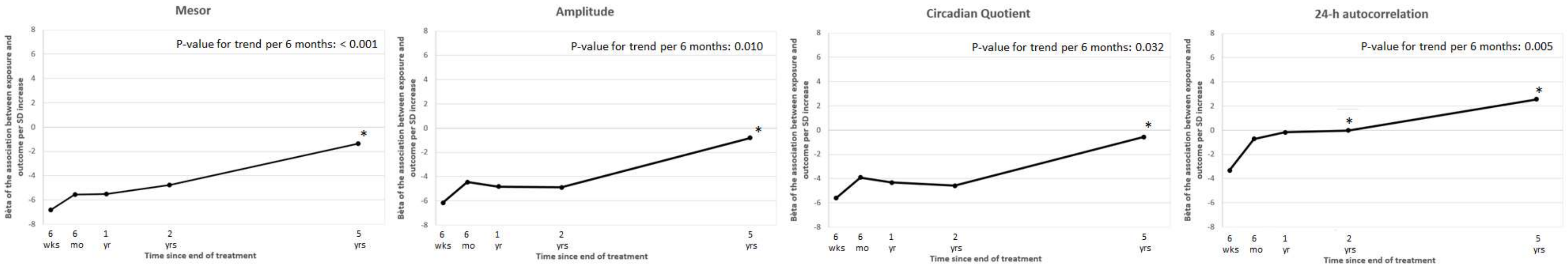

B) CIS – Subjective Fatigue

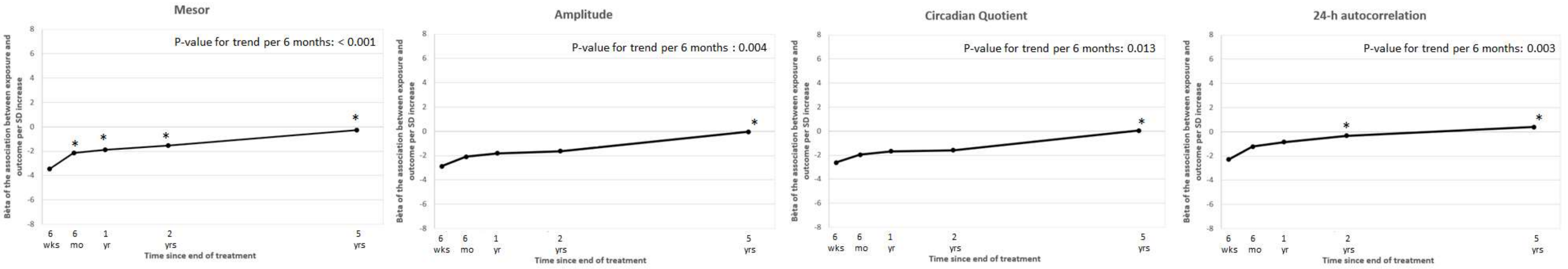

C) CIS – Activity Fatigue

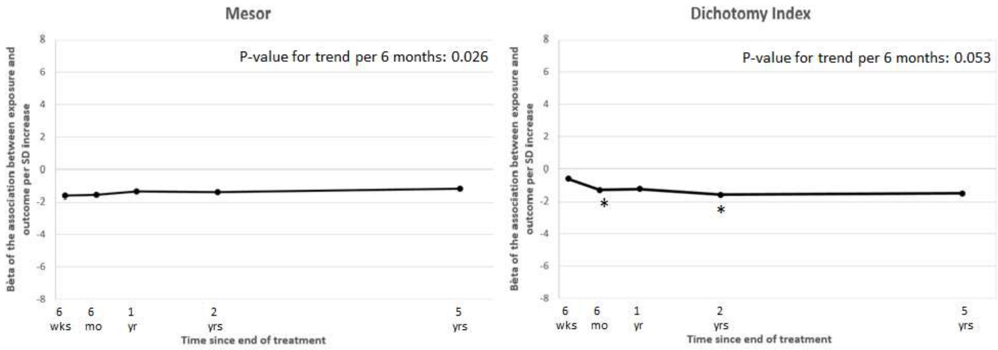

D) EORTC QLQ-C30 – Insomnia (Scale 0 – 100)

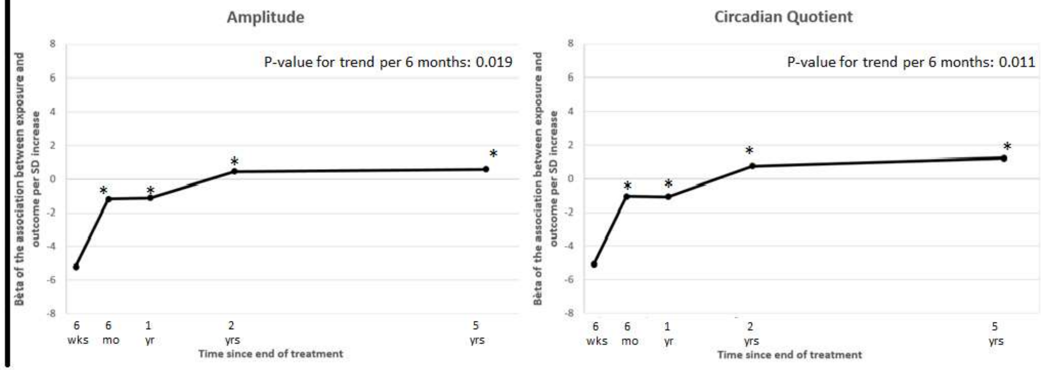

E) EORTC QLQ-C30 – Fatigue (Scale 0 – 100)

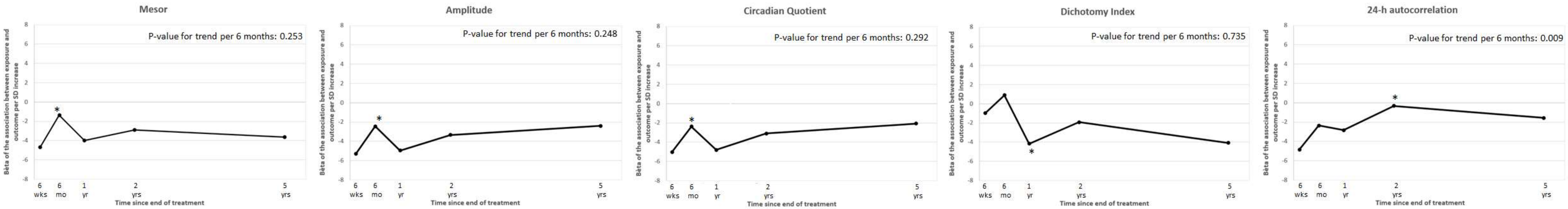

F) EORTC QLQ-C30 – Global Quality of Life (Scale 0 – 100)

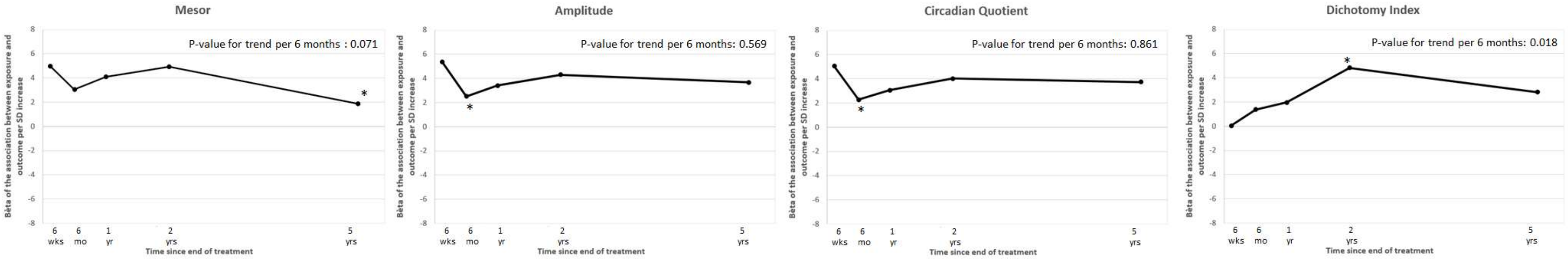

G) EORTC QLQ-C30 – Physical Functioning (Scale 0 – 100)

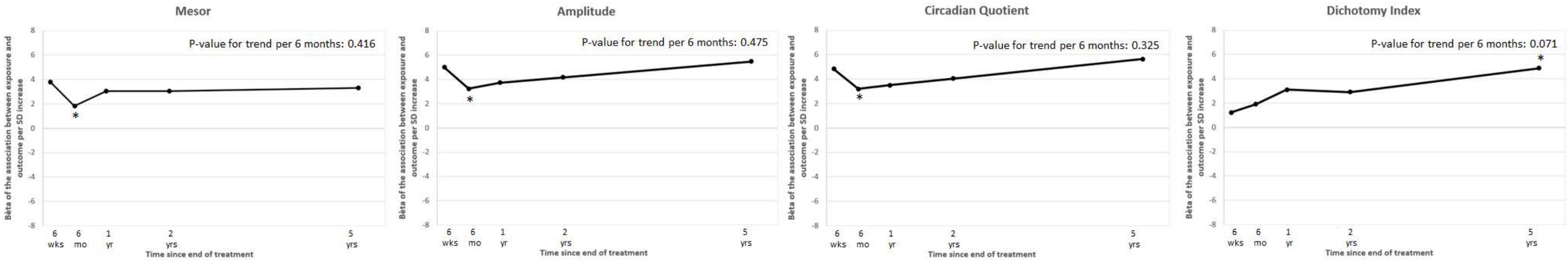

Supplement: Supplementary file 2 — Additional file 2: Supplementary Figure 2. Figure illustrating significant interaction effects of overall longitudinal associations between diurnal rest-activity rhythm parameters with fatigue, insomnia, and HRQoL with time since end of treatment. Stratified effects are shown separately for each post-treatment time point, and significantly different effects as compared to 6 weeks post-treatment are indicated with an asterisk. The P-value for trend indicates if the association between RAR parameters and outcomes differs significantly per 6 months increase in time since end of treatment. In case of no interaction, a horizontal line would be expected. [file 12966_2024_1601_MOESM2_ESM.pdf]
