# Supplementary figures and images for: Longitudinal associations of diurnal rest-activity rhythms with fatigue, insomnia, and health-related quality of life in survivors of colorectal cancer up to 5 years post-treatment
Source: Int J Behav Nutr Phys Act. 2024 May 2;21:51. doi: 10.1186/s12966-024-01601-x (PMC11067118; doi:10.1186/s12966-024-01601-x)

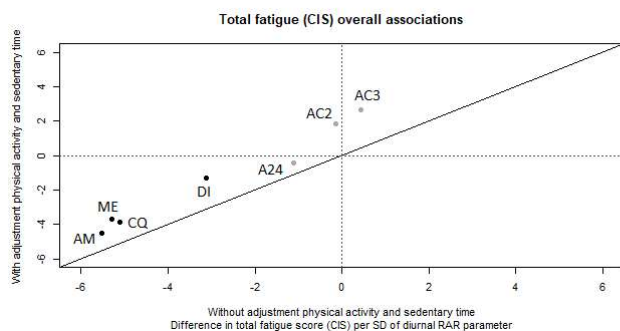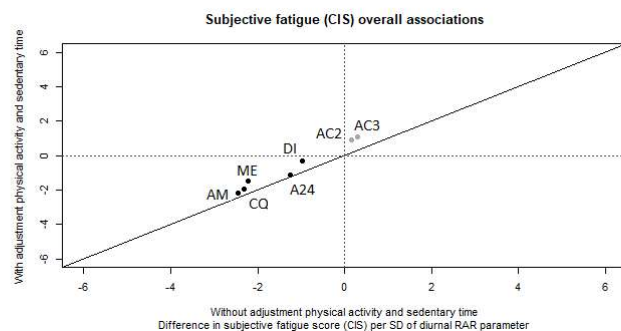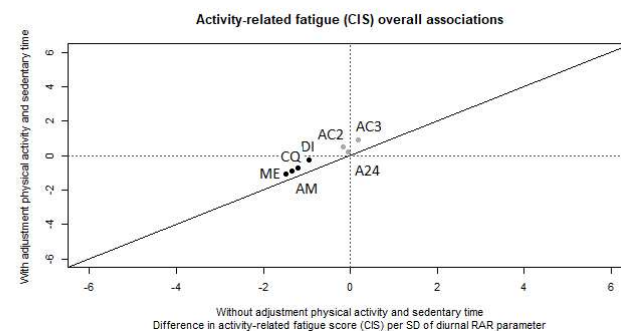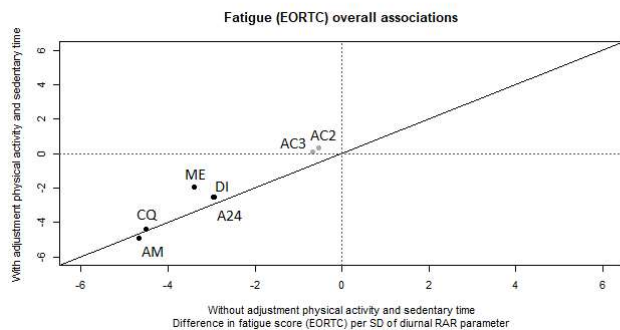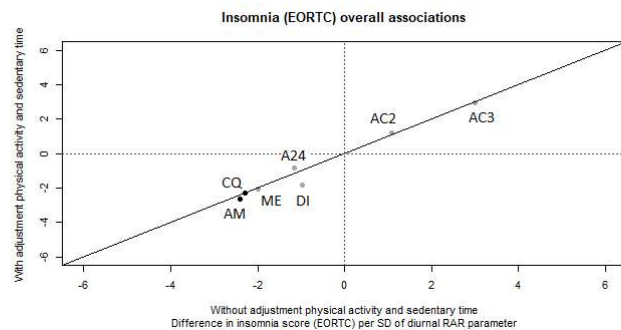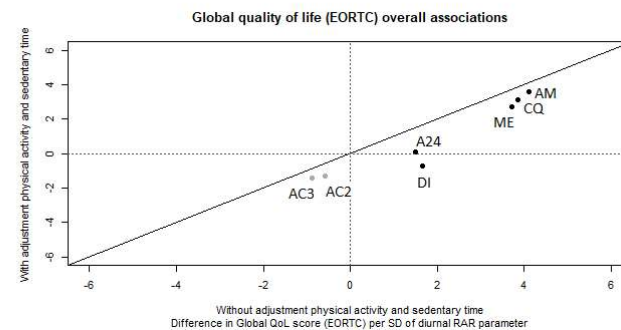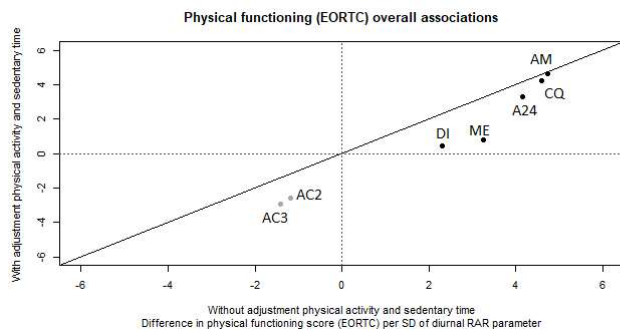

Supplement: Supplementary file 3 — Additional file 3: Supplementary Figure 3. Figure comparing the main results of the overall associations between diurnal rest-activity rhythms parameters and fatigue, insomnia, and HRQoL, and similar associations additionally adjusted for total physical activity and prolonged sedentary time (hours/day). Abbreviations: ME, mesor; AM, amplitude; AC2, acrophase tertile 2; AC3, acrophase tertile 3; CQ, circadian quotient; DI, dichotomy index; A24, 24-h autocorrelation. [file 12966_2024_1601_MOESM3_ESM.pdf]

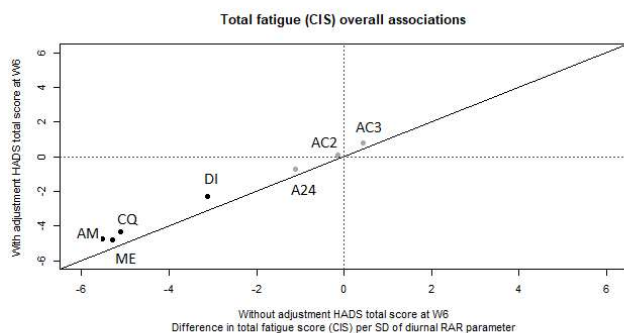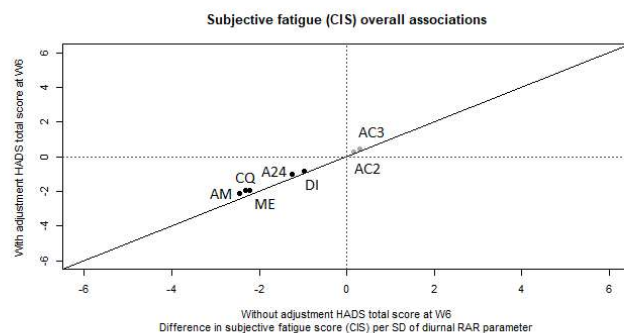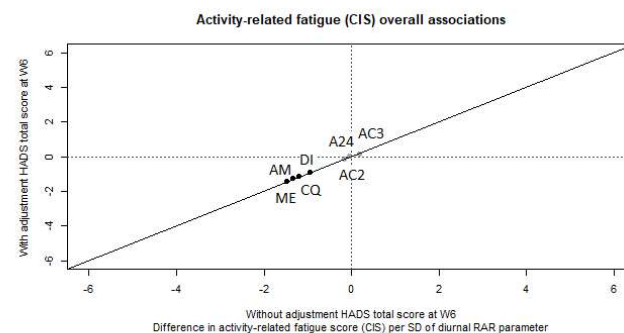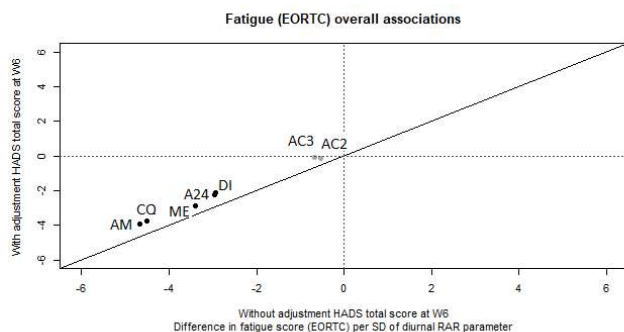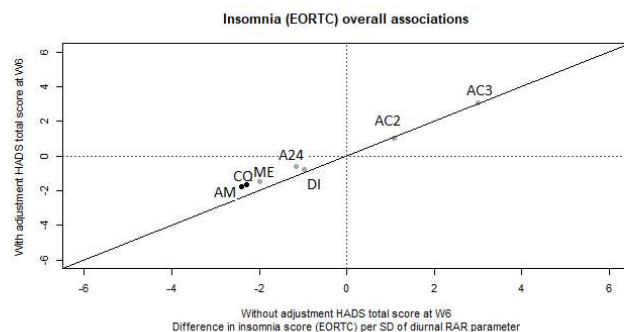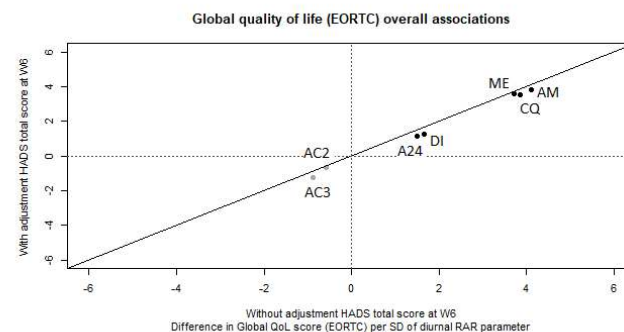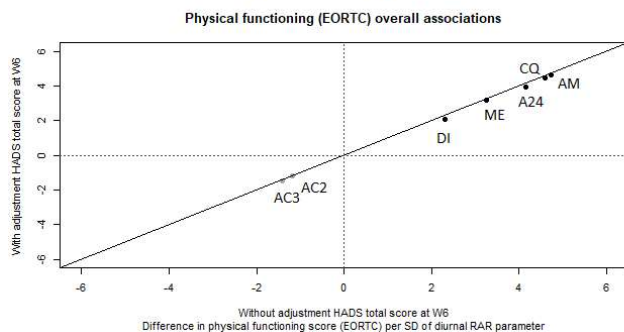

Supplement: Supplementary file 4 — Additional file 4: Supplementary Figure 4. Figure comparing the main results of the overall associations between diurnal rest-activity rhythms parameters and fatigue, insomnia, and HRQoL, and similar associations additionally adjusted for anxiety and depression levels. Abbreviations: ME, mesor; AM, amplitude; AC2, acrophase tertile 2; AC3, acrophase tertile 3; CQ, circadian quotient; DI, dichotomy index; A24, 24-h autocorrelation. [file 12966_2024_1601_MOESM4_ESM.pdf]
