# Supplementary material for: Longitudinal associations of diurnal rest-activity rhythms with fatigue, insomnia, and health-related quality of life in survivors of colorectal cancer up to 5 years post-treatment
Source: Int J Behav Nutr Phys Act. 2024 May 2;21:51. doi: 10.1186/s12966-024-01601-x (PMC11067118; doi:10.1186/s12966-024-01601-x)
Supplement: Supplementary file 5 — Additional file 5: Supplementary Figure 5. Figure comparing the main results of the overall associations between diurnal rest-activity rhythms parameters and fatigue, insomnia, and HRQoL, and similar associations from a time-lag model (exposure at one post-treatment time point combined with outcomes at the next post-treatment time point). Abbreviations: ME, mesor; AM, amplitude; AC2, acrophase tertile 2; AC3, acrophase tertile 3; CQ, circadian quotient; DI, dichotomy index; A24, 24-h autocorrelation. [file 12966_2024_1601_MOESM5_ESM.pdf]

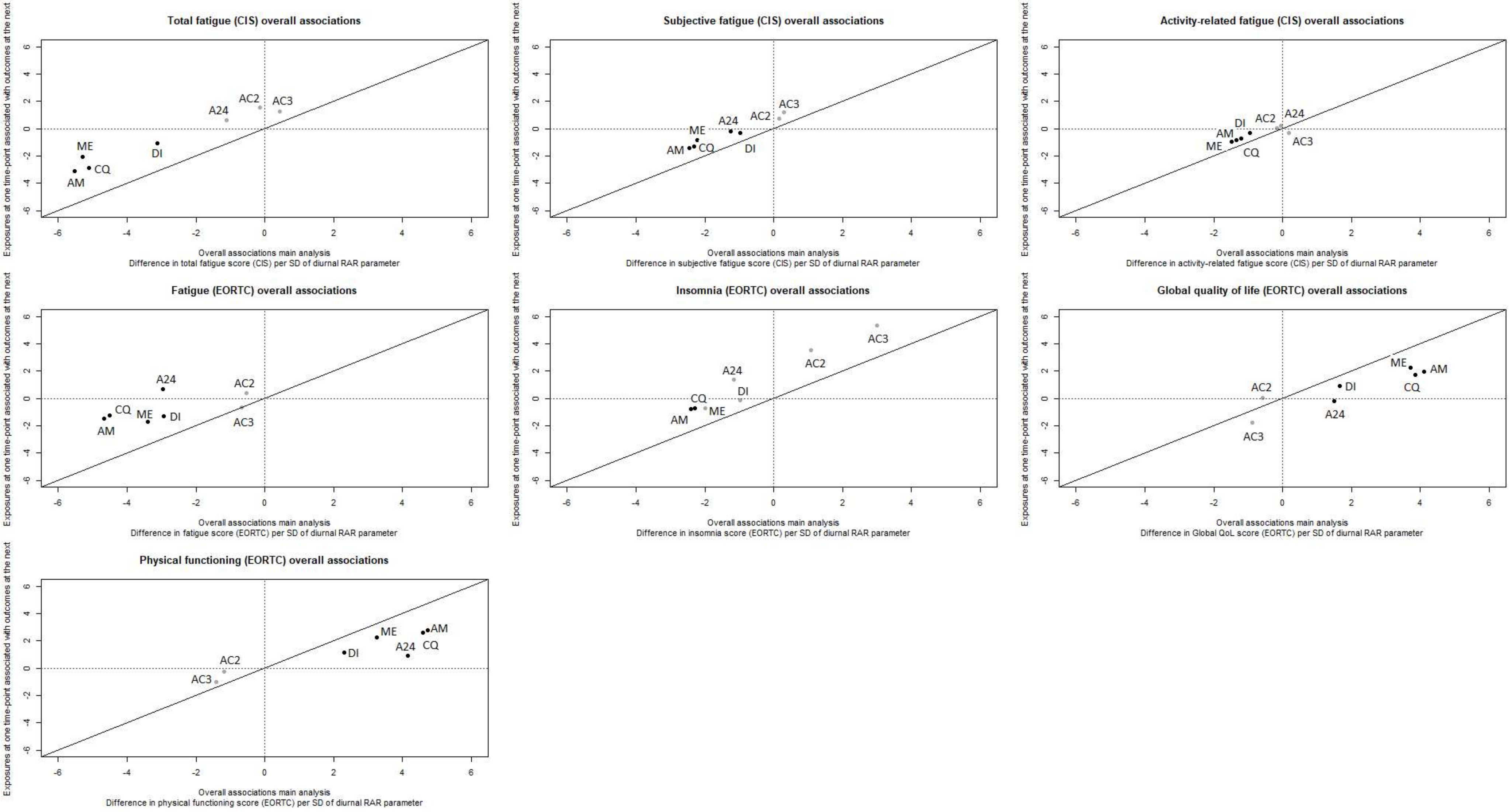

Total fatigue (CIS) overall associations
Subjective fatigue (CIS) overall associations
Activity-related fatigue (CIS) overall associations
Fatigue (EORTC) overall associations
Insomnia (EORTC) overall associations
Global quality of life (EORTC) overall associations
Physical functioning (EORTC) overall associations
Exposures at one time-point associated with outcomes at the next
Overall associations main analysis
Difference in total fatigue score (CIS) per SD of diurnal RAR parameter
Difference in subjective fatigue score (CIS) per SD of diurnal RAR parameter
Difference in activity-related fatigue score (CIS) per SD of diurnal RAR parameter
Difference in fatigue score (EORTC) per SD of diurnal RAR parameter
Difference in insomnia score (EORTC) per SD of diurnal RAR parameter
Difference in Global QoL score (EORTC) per SD of diurnal RAR parameter
Difference in physical functioning score (EORTC) per SD of diurnal RAR parameter
AM
ME
CQ
DI
A24
AC2
AC3
-6
-4
-2
0
2
4
6
