# Supplementary material for: Longitudinal associations of diurnal rest-activity rhythms with fatigue, insomnia, and health-related quality of life in survivors of colorectal cancer up to 5 years post-treatment
Source: Int J Behav Nutr Phys Act. 2024 May 2;21:51. doi: 10.1186/s12966-024-01601-x (PMC11067118; doi:10.1186/s12966-024-01601-x)
Supplement: Supplementary file 6 — Additional file 6. [file 12966_2024_1601_MOESM6_ESM.docx]

**Supplementary Table 1.** Longitudinal associations of diurnal rest-activity rhythm parameter phase difference with fatigue, insomnia, and health-related quality of life in the study population of survivors of colorectal cancer between 6 weeks and 60 months post-treatment.

|  |  | **Checklist Individual Strength (CIS)** | | | **EORTC-QLQ-C30** | | | |
| --- | --- | --- | --- | --- | --- | --- | --- | --- |
| **Phase difference (acrophase – midpoint of sleep), per tertile** | | Total fatigue  (20 – 140) | Subjective  Fatigue  (8 – 56) | Activity  Fatigue  (3 – 21) | Fatigue  (0 -100) | Insomnia  (0-100) | Global QoL | Physical Functioning |
|  |  | *β ^1^* (95% CI) | *β* (95% CI) | *β* (95% CI) | *β* (95% CI) | *β* (95% CI) | *β* (95% CI) | *β* (95% CI) |
| **Phase difference** | Adjusted^2,3^, tertile 1^4^ | 0.5 (-2.5, 3.5) | -0.1 (-1.6, 1.4) | 0.1 (-0.5, 0.8) | -0.7 (-3.4, 2.1) | -0.9 (-4.8, 3.0) | -0.6 (-2.9, 1.7) | -0.2 (-2.1, 1.7) |
|  | Adjusted^2,3^, tertile 2^4^ | REF | REF | REF | REF | REF | REF | REF |
|  | Adjusted^2,3^, tertile 3^4^ | -0.3 (-3.3, 2.6) | -0.2 (-1.7, 1.2) | 0.0 (-0.6, 0.6) | -0.8 (-3.5, 1.8) | 0.1 (-3.7, 3.9) | -1.1 (-3.4, 1.1) | -0.3 (-2.1, 1.6) |

*Abbreviations: CIS, Checklist Individual Strength; EORTC QLQ-C30, European Organization for the Research and Treatment of Cancer Quality of Life Questionnaire.*

*^1^ The β-coefficients indicate the overall longitudinal difference in the outcome score using linear mixed models per tertile of phase difference as compared to tertile 2 (reference).*

*^2^ Linear mixed-models adjusted for sex (male/female), age at enrollment (years), weeks since end of treatment (weeks), neo-adjuvant therapy (yes/no), adjuvant therapy (yes/no), comorbidities (0, 1, ≥2), BMI (kg/m^2^), stoma (yes/no), smoking (former, current, never), employment status (yes/no), and alcohol intake (g/day).*

*^3^A random slope was added to the model when the model improved statistically significantly using a likelihood-ratio test.*

*^4^Tertiles were calculated based on the distribution of values at each individual post-treatment time point. Ranges (clock-hours of phase difference) were as follows: 6 weeks post-treatment, tertile 1 -2.70h – 10.15h, tertile 2 10.17h – 10.91h, tertile 3 10.92h – 17.01h; 6 months post-treatment, tertile 1 7.49h – 10.33h, tertile 2 10.34h – 10.96h, tertile 3 10.97h – 13.15h; 12 months post-treatment, tertile 1 5.62h – 10.27h, tertile 2 10.28h – 10.94h, tertile 3 10.95h – 13.90h; 24 months post-treatment, tertile 1 5.89h – 10.11h, tertile 2 10.12h – 10.88h, tertile 3 10.90h – 13.12h; 60 months post-treatment, tertile 1 8.01h – 10.10h, tertile 2 10.16h – 10.62h, tertile 3 10.72h – 12.23h.*
